# Practical Instruction on Animal Magnetism

**Published:** 1845-04

**Authors:** 


### Art VIII.

1. *Instruction pratique sur le Magnétisme Animal.* Par J. P. F. Deleuze.—*Paris*, 1825.

*Practical Instruction on Animal Magnetism.* By J. P. F. Deleuze.—*Paris*, 1825.

2. *Du Magnétisme Animal en France, et des jugements qu'en ont portés les sociétés savantes, avec le texte des divers rapports faits en* 1784 *par les commissaires de l'Académie des Sciences, de la Faculté et de la Société Royale de Médecine, et une analyse des dernieres séances de l'Académie Royale de Médecine, et du rapport de M. Husson; suivi de considérations sur l'apparition de l'Extase, dans les traitements magnétiques.* Par Alexander Bertrand.—*Paris*, 1826.

*On Animal Magnetism in France, and the Decisions which Learned Societies have made with respect to it, with the Text of the different Reports made in* 1784 *by the Commissioners of the Academy of Sciences, of the Faculty and of the Royal Society of Medicine; and an Analysis of the last Sessions of the Royal Academy of Medicine and of the Report of M. Husson; followed by Considerations on the Manifestation of Extacy in Magnetic Treatment.* By A. Bertrand.—*Paris*, 1826.

3. *Isis Revelata: An Inquiry into the Origin, Progress, and present State of Animal Magnetism.* By J. C. Colquhoun, Esq. Advocate, F.R.S.E.—*Edinburgh*, 1836.

4. *Untersuchungen über den Lebensmagnetismus und das Hellsehen.* Von Dr. Johan Carl Passavant.—*Frankfurt am Main*, 1837.

*Researches on Vital Magnetism and Lucid Vision.* By Dr. J. C. Passavant.—*Frankfort*, 1837.

5. *An Introduction to the Study of Animal Magnetism.* By the Baron Dupotet de Sennevoy.—*London*, 1838.

6. *Histoire Académique du Magnétisme Animal, accompagnée de Notes et de Remarques critiques sur toutes les observations et experiences faites jusqu'à ce jour.* Par C. Burdin, Jeune, et Fred. Dubois (d'Amiens).—*Paris*, 1841.

*Academic History of Animal Magnetism, accompanied with Notes and Critical Remarks upon all the Observations and Experiments hitherto made.* By C. Burdin and F. Dubois.—*Paris*, 1841.

7. *Facts in Mesmerism, and Thoughts on its Causes and Uses.* By Charles Caldwell, M.D.—*Louisville*, 1842.

8. *The Zoist: A Journal of Cerebral Physiology and Mesmerism, and their applications to Human Welfare.*—*London*, 1843-4.

9. *Neurypnology; or the Rationale of Nervous Sleep, considered in relation with Animal Magnetism. Illustrated by numerous Cases of its successful application in the Relief and Cure of Disease.* By James Braid, M.R.C.S.E. M.W.S. &c.—*London*, 1843.

10. *Manuel Pratique de Magnetisme Animal; exposition méthodique des procédés employés pour produire les phénomènes magnétiques, et leur application à l'étude et au traitement des maladies.* Par Alph.

Teste, Docteur en Médecine de la Faculté de Paris, Membre de plusieurs sociétés savantes.—*Paris*, 1843.

*Practical Manual of Animal Magnetism, with a Methodical Exposition of the Processes employed to produce the Magnetic Phenomena, and their application to the Study and Treatment of Disease.* By A. Teste.—*Paris*, 1843.

11. *Facts in Mesmerism, with Reasons for a dispassionate Inquiry into it.* By the Rev. Chauncy Hare Townsend, a.m. late of Trinity Hall, Cambridge.—*London*, 1844.

12. *Letters on Mesmerism.* By Harriet Martineau.—*London*, 1845. 12mo.

13. *Medical Report of the Case of Miss H—— M——.* By T. M. Greenhow, Fellow of the Royal College of Surgeons of England, &c. —*London*, 1845. 8vo, 1845.

14. *Human Magnetism; its claims to dispassionate inquiry. Being an attempt to show the utility of its application for the relief of human suffering.* By W. Newnham, m.r.s.l. &c.—*London*, 8vo, 1845.

We do not rank with those who would decry the investigation of any probable or possible truth, because it may have sustained the admixture of error or folly, or because it may have been associated with charlatanry and deceit. Almost every branch of knowledge has, at one period of its history, been subjected to some degrading alliance. Astronomy, for ages, was yoked with astrology; chemistry was little more than the handmaid of alchemy; and practical medicine has ever been degraded by the wickedness and imposture of some of its professors. Such a state of things may be observed in well-nigh every department of human inquiry. The records of history abound in falsehood and exaggeration; and subjects the most sacred have been and are disfigured and perverted. A combination of error and truth, indeed, would seem to characterize most matters on which the human understanding exercises itself. Let this unhappy association, however, be as intimate as it may, we conceive that there is thereby furnished no just motive for rejecting the truth because of the error. It is for us rather to examine, search into, and try to determine the actual amount of the true, whereby we attain a better position from which to reject, and confute, and render powerless, the false.

It is with these sentiments that we propose to ourselves a brief investigation of the existing pretensions of Animal Magnetism, or Mesmerism. In proceeding to prosecute this task, we shall, in the first place, advance the reasons which, in our judgment, urge a dispassionate examination of the subject; secondly, we shall point out the kind of evidence we deem necessary for the establishment of each class of the alleged phenomena; and we shall then proceed to discuss the question of their validity.

It is Bossuet, we believe, who somewhere affirms that every error is a truth abused; and it has been most wisely said, that commonly there is to be found a truth at the bottom of any *honest* extravagance. Would it seem unreasonable, to believe that the same observations might apply to the subject, the discussion of which we now approach? Is not Animal Magnetism, however encompassed with error, the abuse of a truth rather than an absolute fiction? And, whilst contemplating the huge pile of nonsense, fancy, and fable, to which writers on this subject direct our attention, many of them in good faith, may it not be possible to detect some truth at the base? We hesitate not to assert our own conviction that it is so; and in the course of this article, we shall develope the facts and the reasonings which have led us to such a conclusion.

We think that mesmerism has hardly received fair play at the hands of many of our professional brethren, or in the pages of some of our contemporaries. Its pretensions, to some extent, however, are too well supported both by the number and the respectability of the witnesses, to justify an opposition made up almost exclusively of ridicule and contempt. Preposterous enough, doubtless, are many of the statements we are doomed to encounter in the perusal of mesmeric books; yet some of the facts recorded in these publications, and seemingly well authenticated, possess a wide range of interest, regarded in many points of view. But, independently of this, when it is considered, that men like Cuvier, Laplace, Hufeland, and Treviranus, have not refused their testimony as to the reality of some of the facts of mesmerism, we hardly think it right to dispose of the whole question, unexamined, by the facile process of a self-complacent *poo-poo!* It is true that contempt is the feeling naturally excited by the mighty airs of effrontery with which the ultra-mesmerists do, at times, advance even their most outrageous propositions, with taunts, ever ready, about persecution, Harvey, and Galileo! Nevertheless, we think it better, as a matter both of justice and policy, to disregard all the superimposed folly and imposture, and dispassionately to investigate the actual physiological facts and their true relations, with a view to discover, if it be possible, their nature, the extent of their novelty, and the practical inferences to be legitimately deduced from them.

We ourselves entertain not the slightest bias or prejudice upon either side of the question. We have at no time resolved that the thing could not, or should not, be so.* We have never pursued or examined the

* The following extract from the concluding portion of our former article on Animal Magnetism, published six years since, (No. XIV, April, 1839,) will show the spirit in which we have always viewed this subject. "Our object has not been to give expression to our feelings, but to present to the reader's consideration an historical record, which may be reflected upon with some benefit. Neither would we be so far influenced by the impostures occasionally practised under the name of magnetism, as wholly to deny that some of the phenomena, from time to time produced by all aspirers to the art, seem to result either from some principle heretofore unknown and not yet correctly designated, or from some modification of recognized principles in the animal economy which cannot yet be accurately limited or defined. The whole of man's existence is too mysterious, and he is surrounded by too many things utterly beyond his comprehension, to justify an obstinate disbelief of things hard to be understood. In the constant attempts of the human intellect to penetrate the thick curtain that hangs all around it, doubtless some transitory glimpses of hidden truths are now and then accorded to quick intellects and peculiar organizations; and there is ever much more in heaven and earth 'than is taught in our philosophy.' The temporal guides of man, however, are his senses and his reason; and when he lays claim to a wisdom and to powers which are incapable of being made palpable to the one or explicable to the other, although we may not presume to say that he cannot possibly be right, he must expect that we make very diligent use of our own senses and our own reason in the investigation of his evidence; and industriously endeavour to untwist the double chain of truth and fancy, which he would fain twine round our puzzled understandings."

subject with an appetite predisposed by wonder for the too-ready reception of novelty; and, at the present moment, if we rightly appreciate our own mental condition, we have no *feeling* whatever upon the subject. Within the last few years, we have at various times dipped into mesmeric literature, and we have also witnessed a reasonable amount of mesmeric facts; but we have never gained a prepossession by prosecuting the matter as a *special study;* nor, indeed, have we instituted, excepting incidentally, any experiments of our own. We have, simply, as dispassionate lookers on, as unprejudiced observers of what has fallen in our way, attempted the formation of an accurate judgment upon what we have seen and read; and our conclusions, in the sequel, we shall lay before the reader.

The facts of mesmerism, as set forth by systematic writers, fairly permit of their division into two very distinct classes: one includes phenomena which, if true, do not violate any recognized analogy of nature; and the other comprehends circumstances which contravene, seemingly at least, every admitted experience of the past and the general judgment of mankind. Instances of the former kind—the *ordinary* manifestations of mesmerism—are represented as being of frequent occurrence, and as almost producible at pleasure, and thereby accessible to every inquirer; so that, with respect to their reality, any competent observer may come to a decision by personally examining them; and to this course, where practicable, every one is plainly bound, before an opinion upon the subject can justly be given. The *extraordinary* phenomena are stated to be comparatively rare, and not to be generally accessible; and, in the investigation of these, a scrutiny of the evidence is usually the only resource which is left.

In dealing with the testimony relating to any branch of this subject, the rule, of course, is to be followed which would be deemed admissible in the investigation of any other philosophical question. The method of induction can alone lead to satisfactory results. The nature of the individual facts must be accurately appreciated, their authenticity must be carefully sifted, and the mental character of the witnesses must be rightly understood; in a word, correct premises must supply the materials for truthful inference. We do not think that mesmerism can justly be dealt with by a mere appeal to principles already admitted. So long as any proposition comes recommended to us in a manner implying no actual contradiction to some known fact, we do not see how any supposed natural law can philosophically enforce its rejection, even should it seem directly opposed to principles hitherto regarded as fixed and established. A *law of nature* can only be deduced by observation of natural operations; and when any novel fact arises, opposed to what had previously been deemed a fixed principle, the terms which express it must sustain modification, and not the fact itself receive a denial. But, maintaining this position most rigorously, we must insist upon the rule that evidence, tendered in support of whatever is new, must correspond in strength with the extent of its incompatibility with doctrines generally admitted as true; and that where statements obviously contravene all past experience, and the almost universal consent of mankind, any evidence is inadequate to the proof, which is not complete, beyond suspicion, and *absolutely incapable of being explained away.*

We hold that, here, it is not necessary always to *prove* the accuracy of an explanation offered; the facts of the case are not always cognizable; it is enough that, in the case supposed, some phenomenon shall *allow* an interpretation which brings it into harmony with antecedent experience, for the opposite view to be rejected as *unproved.* Guided by these canons, we shall proceed to examine the pretensions of mesmerism, with a view to attain some accurate conception of the existing state of the question, and of the estimation in which it should be held by the members of our profession.

It must be kept in mind that some of the asserted facts of mesmerism are distinct from, and do not necessarily lead to, the theories invented to explain them; that is to say, certain reported phenomena may be real, and yet the notion of animal magnetism be unsupported. Before we conclude, each of these points shall receive consideration; our first object, however, before discussing the question of *specific* influence in mesmerism, will be an analysis of the statements made with respect to the facts, and an attempt to make out the physiological or pathological character appertaining to them.

The effects following the mesmeric processes are various, according to the constitutional temperament of those upon whom trials are made; the most susceptible being, most commonly, young women whose nervous systems are mobile and *impressionable.* It is set forth on all hands that in a great majority of instances, no apparent result follows; and that, of the minority susceptible, a considerable number experience little more than drowsiness, or common sleep. Sometimes, however, there is said to supervene a state of coma; at others, exaltation, depression, or some anomalous modification of sensibility; and, occasionally, a state somewhat approaching to that of reverie, wherein the individual, although conscious, feels incapable of independent exertion, and spell-bound, as it were, to a particular train of thought or feeling. The occurrence of convulsive action, and of muscular rigidity, is described as taking place in some cases to a greater or less extent. These results are said to constitute the *simpler phenomena* of mesmerism. We shall illustrate them by some extracts from accredited writers upon the subject.

"In this peculiar state of sleep, the surface of the body is sometimes acutely sensible, but more frequently the sense of feeling is absolutely annihilated. The jaws are firmly locked, and resist every effort to wrench them open: the joints are often rigid, and the limbs inflexible; and not only is the sense of feeling, but the senses of smell, hearing, and sight, also, are so deadened to all external impressions, that no pungent odour, loud report, or glare of light, can excite them in the slightest degree. The body may be pricked, pinched, lacerated, or burnt; fumes of concentrated liquid ammonia may be passed up the nostrils; the loudest reports suddenly made close upon the ear; dazzling and intense light may be thrown upon the pupil of the eye; yet so profound is the physical state of lethargy, that the sleeper will remain undisturbed, and insensible to tortures, which, in the waking state, would be intolerable." (Dupotet, p. 36.)

The above concise sketch corresponds very closely with what is laid down in other works of mesmeric repute. A few brief quotations exhibiting this correspondence we subjoin. The first we take from Deleuze's 'Practical Instructions,' wherein he states that "the magnetisee feels the necessity of closing the eyes; his eyes are so sealed that he cannot open them; he experiences a calm, a feeling of comfort; he becomes drowsy; he is put to sleep." Teste, another writer of distinction, speaking of the physical insensibility, says, "it exists, not only in the skin, but in the subcutaneous tissues, in the muscles, and even in the nervous ramifications." Dr. Passavant of Frankfort, an author often referred to, avers as follows: "As an especial effect of the power of animal magnetism, results the magnetic sleep. This is mostly deeper than ordinary sleep, the mediation of the senses is yet more decidedly suspended. The sensibility can so have vanished in a moment, that the loudest sound, the brightest light, even bodily injuries, are not perceived in this sleep." Indeed, all the authorities seem to coincide very much in their accounts; and, this we say, after referring to Chenevix, Elliotson, Townshend, Gauthier, Foissac, and others.

In mesmeric literature, innumerable records of cases will be found exemplifying what has just been adduced. We shall here extract the particulars of two or three of these, as being at once among the most striking and the best authenticated. In the 'Lancet' for December 9th, 1837, the following account appears from the pen of Dr. Sigmond:

"I was enjoying the hospitality of a most amiable family in Fitzroy square, when animal magnetism became the topic of conversation, *and I related the trials I had already made. One of the young ladies proposed to become the subject of experiment*, to which I very willingly assented; for, having on former occasions attended her during momentary sickness, I was fully aware of the natural strength of her constitution, and the absence of that nervous temperament which renders this system totally inapplicable. I began what are technically called 'the passes.' They, as is not unusual, excited laughter and incredulity. I proceeded for about five minutes, and then stopped and *inquired if any sensation were produced*, and the answer was 'a slight sleepiness,' and ridicule was again thrown upon the subject. I recommenced the manipulations; I observed the eyelids falling, and at last they closed; but, as the same incredulous smile remained, I persevered for three or four minutes, when I, almost doubting whether any influence had been produced, inquired what the feelings were, to this no answer was returned. I found my young friend was in the most complete trance I had ever witnessed as the result of my magnetism. The stupor was the most profound; and I then tried the usual means to arouse her, but they were vainly exercised. After a few minutes, I found the hands become icy cold, the face lost its natural hue, and became perfectly pallid; the extremities became quite cold; the respiration was imperceptible; the stimulus of light did not affect the eye; on speaking to her, a faint smile was excited, and a quivering of the lower jaw, which seemed to indicate a wish but an incapacity of answering; the pulse became gradually feebler, whilst the external appearance altogether bore such a decidedly deathly cast, that naturally some apprehension was excited amongst her family by whom she was surrounded. I placed the perfectly unconscious subject of this distressing scene in a horizontal position, and directed the application of warmth and of friction to the extremities. Circulation and animal heat were gradually excited, but she presented a most singular appearance of suspended animation. In this condition she remained more than four hours, for I had commenced a little after ten in the evening, and it was about half-past two, that, on some slight effort being made to rouse her, she uttered some of the most piercing shrieks I have ever heard; there were convulsive efforts to raise the limbs; the face, too, became convulsed; she opened her eyes and stared wildly around; she was placed in the upright posture, and seemed sensible. Advantage was taken of this circumstance to carry her to her apartment; before, however, she could reach it, she fell into a profound slumber, but its character was more natural. She was placed in her bed, appearing perfectly composed; the countenance had acquired its natural hue; the respiration was perfectly easy, and the pulse natural. In this state, she remained during the whole of the day, until nine o'clock in the evening, once only opening the eyes, and addressing a few words to an anxious and affectionate sister. In the evening, the young lady joined her family perfectly restored to her wonted cheerfulness. She expressed no complaint whatever."

Two recent histories, most probably fresh in the memory of some of our readers, constitute striking illustrations of alleged physical insensibility, so much dwelt upon by writers on the subject of our present discussion. We refer to the cases of amputation of the thigh during the mesmeric sleep, set forth as having occurred without the knowledge of the patients, who, during the operation, were stated by themselves, and considered by others, to have experienced no sensation whatsoever. The first of these occurred in the practice of Mr. Ward, of Wellow in Nottinghamshire, in the month of October, 1842, and is authenticated by himself, and Mr. Topham, a barrister of the Temple. Some particulars of this case we proceed to give. It was described by these gentlemen, in a paper readbefore the Royal Medico-Chirurgical Society, and afterwards published as a pamphlet, as "one of very extensive ulceration of the cartilages of the knee-joint, of four and a half years' standing; the consequence of neglected inflammation of the synovial membrane." We shall enter into no detail of the preliminary treatment here pursued, but shall go at once to the account furnished of the amputation, ultimately decided upon: the statement is in the language of Mr. Topham, who acted as mesmeriser in this instance.

"At half-past one o'clock, we proceeded to Wombwell's room, to make the necessary arrangements. From the suffering inflicted by the slightest movement, it was found impossible, without needless torture, to place him upon a table. The low bed, on which he then lay; was therefore lifted upon a temporary platform. Ten minutes after being mesmerised, he was drawn, by means of the bed clothes beneath him, towards the end of the bed. The movement, however, excited that pain which had so often aroused him before; and now it did so again. There was something quite excruciating in the suffering which the state of the knee produced; for I had seen him, whilst in mesmeric sleep, pricked to some little depth, in other parts of the diseased limb, without being disturbed or conscious of it. To preclude the necessity of any further movement, his leg was now placed in the most convenient position which he could bear. Shortly afterwards, he declared that the pain had ceased; and I again mesmerised him, in four minutes. In a quarter of an hour, I informed Mr. Ward that he might commence the operation. I then brought two fingers of each hand gently in contact with Wombwell's closed eyelids; and there kept them, still further to deepen the sleep. Mr. Ward, after one earnest look at the man, slowly plunged his knife into the centre of the outside of the thigh, directly to the bone; and then made a clean incision, round the bone, to the opposite point, on the inside of the thigh. The stillness, at this moment, was something awful: the calm respiration of the sleeping man alone was heard; for all other seemed suspended. In making the second incision, the position of the leg was found more inconvenient than it had appeared to be; and the operator could not proceed with his former facility. Soon after the second incision, a moaning was heard from the patient, which continued, at intervals, until the conclusion. It gave me the idea of a troubled dream; for his sleep continued as profound as ever. The placid look of his countenance never changed for an instant; his whole frame rested, uncontrolled, in perfect stillness and repose; not a muscle or nerve was seen to twitch. To the end of the operation, including the sawing of the bone, securing the arteries, and applying the bandages,—occupying a period of upwards of twenty minutes,—he lay like a statue. .... Finally, when all was completed, and Wombwell was about to be removed, his pulse being still found very low, some sal volatile and water was administered to him; it proved too strong and pungent, and he gradually and calmly awoke.

"At first, he uttered no exclamation; and, for some moments, seemed lost and bewildered; but, after looking round, he exclaimed, 'I thank the Lord to find its all over!' He was then removed to another room; and, following immediately, I asked him in the presence of those assembled, to describe all he felt or knew, after he was mesmerised. His reply was, '*I never knew anything more; and never felt any pain at all; I once felt as if I heard a kind of crunching.*' I asked him if that were painful? He replied, '*No pain at all! I never had any, and knew nothing, till I was awakened by that strong stuff,*' (the sal volatile.) The 'crunching,' no doubt, was the sawing his own thigh-bone."

These statements are fully borne out by the written account of Mr. Ward himself, appended to that of Mr. Topham; and the case, so authenticated, received at the time a variety of corroboration from other quarters. Before we remark on the credibility of this narrative, we shall supply the leading particulars of the second case, which occurred in the practice of Mr. Toswill, of Leicester. The account we subjoin is from the 'Leicestershire Mercury,' of August 31st, 1844, and copied by us from the 'Zoist' of the succeeding October.

"The patient is a young woman of the name of Mary Ann Lakin, 16, Fleet-street, in this town, who had been afflicted with a disease of the knee-joint for four years. The precise nature of the disease we do not know; but it was attended with enormous swelling of the limb, and with such excruciating pain as to prevent anything like consecutive rest for a long period............ As amputation of the diseased joint was deemed essential by her medical attendant, it was decided that the operation should be performed while in the mesmeric state. Accordingly, twelve o'clock on Thursday morning was fixed upon for the operation to take place. Mr. Hollings was the mesmeriser, and Mr. Toswill the operator, besides whom were present Dr. Shaw, and Messrs. Paget, Seddon jun., Downing, &c. Mr. Hollings having mesmerised the patient, which was accomplished in about nine minutes, Mr. Toswill proceeded to perform the operation. The limb was taken off within about five inches of the hip-joint, the spot measuring thirty-three inches in circumference where the amputation took place, and which was effected in two minutes and a half. During the operation an all but inaudible moaning was heard, and a slight movement of the body was perceptible; but, as far as could be judged, there was an entire absence of pain. This was evinced by the countenance preserving throughout the greatest placidity, not a single motion of a muscle indicating such sensation. On being demesmerised, the patient was not aware what had taken place, till informed by those in attendance."

The above statements have been further detailed and substantiated in the written accounts of Mr. Toswill, Mr. Downing, and Mr. Hollings. Dr. Shawe and Mr. Paget demurred somewhat to the conclusion, that the operation had been painless; all, however, concurred in the statement, that the patient *professed* to have felt no pain. There ensued some controversy on the matter, which may be seen in the number of the *Zoist* from which our extract has been taken. A perusal of this controversy, however, will not remove the impression that there did, very probably, exist some remarkable modification of sensibility in this instance.

Facts in many respects analogous to the above we have ourselves frequently witnessed. We have, for instance, seen great nervous disturbance, apparent sleep, and suspension of sensibility to occur under the mesmeric operations; and, with every disposition in the world to ascertain any existing deception, we have failed in the attempt. We have said that we have seen the induction of insensibility; at any rate, we have witnessed an entire absence of all indications of feeling under circumstances most likely to call them forth. We have seen a needle thrust deeply under the nail of a woman sleeping mesmerically, without its eliciting a quiver; we have seen pungent snuff in large quantities passed up the nostrils, under the same circumstances, and no excitement produced, until the patient was roused, many minutes afterwards; we have noticed an immunity from all shock, when percussion-caps have been discharged suddenly and loudly close to the ear; and we have observed a patient's little finger in the flame of a candle, and yet no indication of pain. In this latter case, all idea of there having been courageous dissimulation was removed from our mind in seeing the same patient afterwards evince both surprise and indignation at the treatment received; as, from particular circumstances, a substantial inconvenience was to result from the injury to the finger, which was by no means slight.

Now, that some such results as those which have been exemplified in the accounts of Dr. Sigmond, Messrs. Ward and Topham, and Mr. Toswill, and in those supplied by our own experience, do now and then really follow the mesmeric processes, it is, in our opinion, a much easier matter to allow, than to explain how different men, of acknowledged high character, of different places, at various times, and with every motive for guarding their own reputation jealously, should either be themselves so utterly deceived in a matter of simple observation, or, still more, should coincide in the intention to deceive the world upon this subject. It may, indeed, be contended that the statements themselves are of so extraordinary a character as to be absolutely incredible, even on the testimony of one's own senses. But this is a conclusion so outrageous, that it can be only justified by proof of the most incontrovertible kind. Before admitting its justice, we shall therefore subject the matter in question to the method of investigation proposed a few pages back; inquiring, in the first instance, into the nature of the asserted phenomena, with a view to determine the extent of their novelty, or incompatibility with past experience; and then attempting to decide as a matter of evidence, the question of their truth or falsehood.

Every one conversant with practical medicine knows well enough that a vast variety of bodily derangements occur, the chief seat of which would appear, from the symptoms, to be in the nervous system, of a character little appreciable as to their intimate nature, and upon which morbid anatomy sheds no light; we allude to that anomalous class of maladies commonly represented by the term HYSTERIA. It is true that, from the comparative frequency of certain forms of hysterical disease, some pathologists would persuade themselves that it is not, after all, so very obscure in its pathology, but that we really do know something definitive about the whole matter; but any one who shall consult the writings of the most practical and scientific will see nothing but discrepancy and dispute; and it will be found that some of the soberest heads virtually acknowledge that we know indeed very little upon the subject. Now, in this capacious, ill-understood group of diseases, will be found constantly included cases in close correspondence—judging from their recorded symptoms—with the mesmeric examples given in the foregoing pages; and a like correspondence will be noticed in the general descriptions of this class of diseases supplied in standard writings on the subject. Thus, Dr. Conolly, in his classical treatise published in the 'Cyclopædia of Practical Medicine,' states that, "in some cases, the hysteric fit consists of temporary or partial loss of power, or a palsy of some of the voluntary muscles—sometimes of those of the voice, and sometimes of all the voluntary muscles of the body, and the patient falls into a state of coma; is insensible; the face is flushed; the pulse beats regularly, even firmly; the respiration is calm and profound; and neither the sensibility nor the power of voluntary motion returns for several hours." Let the following from the same treatise be compared with the alarming features displayed in Dr. Sigmond's case: "The functions of the heart and lungs may be so seemingly suspended, and the coldness so great as to present the image of death;" further on, "there may be rigid spasm of several of the muscles, especially of the limbs." In the article, *Hysteria*, in Dr. Copland's 'Dictionary,' it is observed, "in a few instances the paroxysms change their character, and assume the form of *catalepsy*;" and, in treating of this latter functional aberration, Dr. Copland says, "in the more complete seizures, sense, intelligence, voluntary motion, and consciousness are entirely abolished; but, in some instances, the abolition is only partial; the patient being conscious, but incapable of moving or speaking."

Who, vaguely acquainted with mesmeric literature, would not suppose, in reading these extracts, that we were quoting from some of the works, the titles of which we have prefixed to this article? The accounts, for the most part, will apply alike to a large proportion of mesmeric cases, and to the forms of disease discussed by these learned authors, and more or less familiar to every practitioner.

It would unnecessarily preoccupy our assigned limits, to adduce many instances in illustration of what we have taken from Drs. Conolly and Copland. We will, however, supply some particulars of three or four, which will be allowed to exhibit much similarity with the cases of the mesmerists. The following example of what we will term, for want of a better designation, *hysteric* trance is related by Dr. G. M. Burrows, in his 'Commentaries on the Causes, &c. of Insanity;' in its leading features, it very much resembles the instance of *mesmeric* trance, related by Dr. Sigmond. It was the case of a young woman in whom the catamenia having been suddenly suppressed, a state of active mania ensued, on the subsidence of which she fell into the condition described below:

> "She became a perfect statue; sensation and volition were quite suspended; the evacuations were discharged involuntarily; mouth open, and saliva flowing from it in large quantities; a constant sardonic grin; the eyes immovable and imbedded in the upper eyelids; every limb retained the position in which it was placed, even the most painful, and that for a time impossible to be preserved by any one in health. The pulse was soft and slow........... At last, she rose one morning, in possession of every faculty, mental and bodily. She voluntarily assisted in domestic affairs, and talked rationally and cheerfully. She had a perfect recollection of most things prior to the attack of catalepsy; but, from the accession of that affection, all was a blank."

The above account is taken from one of Dr. Laycock's papers on hysteria, published in the 49th volume of the 'Edinburgh Medical and

Surgical Journal;' and a case very similar in many respects is related by Dr. Gooch, in the 'Medical Transactions:' an abstract of the paper occurs in Good's 'Study of Medicine,' from which the subjoined extract is taken. The symptoms displayed themselves in the puerperal state; the patient having, a few days before, given birth to a dead child. The following is in the language of Dr. Gooch:

"A few days after our visit, we were summoned to observe a remarkable change in her symptoms. The attendants said she was dying, or in a trance. She was lying in bed, motionless, and apparently senseless. It had been said that the pupils were dilated and motionless. But, on coming to examine them closely, it was found that they readily contracted when the light fell upon them; her eyes were open, but no rising of the chest, no movement of the nostrils, no appearance of respiration could be seen; the only signs of life were her warmth and pulse; the latter was, as we had hitherto observed it, weak, and about 120; her fæces and urine were voided in bed.

"The trunk of the body was now lifted, so as to form rather an obtuse angle with the limbs (a most uncomfortable posture), and there left with nothing to support it. Thus she continued sitting, while we were asking questions and conversing, so that many minutes must have passed.

"One arm was now raised then the other, and where they were left, there they remained; it was now a curious sight to see her, sitting up in bed, her eyes open, staring lifelessly, her arms outstretched, yet without any visible signs of animation; she was very thin and pallid, and looked like a corpse that had been propped up, and had stiffened in this attitude. We now took her out of bed, placed her upright, and endeavoured to rouse her by calling loudly in her ears, but in vain; she stood up, but as inanimate as a statue; the slightest push put her off her balance; no exertion was made to regain it; she would have fallen, if I had not caught her.

"She went into this state three several times. The first time it lasted fourteen hours; the second time, twelve hours; and the third time, nine hours; with waking intervals of two days after the first fit, and one day after the second." (Vol. iv, p. 614. 3d edit.)

With respect to the two cases of amputation recorded as having taken place during mesmeric coma, we shall not insist upon the fact, that the patients sustained the loss of limb in a state of *perfect* unconsciousness, although we imagine that it will very generally be admitted that there did exist some unwonted depression of sensibility. Assuming, however, that a degree of coma did really obtain in those cases, the following instance will constitute their analogue, and that also of many other mesmeric facts; it is related by Dr. Cooke, in his 'Treatise on Nervous Diseases:'

"A lady about 20 years of age, who had usually enjoyed very good health, was one morning found in a state of profound but quiet sleep, from which she could not be awakened, although the preceding evening she had gone to bed apparently quite well. Various means had been tried, with a view of exciting her from this state, but in vain. Under these circumstances, I recommended cupping in the neck; and after she had lost a few ounces of blood in this way, she opened her eyes, perfectly recovered, and remained through the day quite free from all symptoms of disorder. The next morning, and for several successive mornings, she was found in a similar state, from which she was recovered by the same remedy, no stimulating external applications producing any good effect. As she was considerably weakened by repeated depletions, it was determined that, on the next recurrence of the paroxysm, the case should be left to the efforts of nature, as long as was consistent with safety. The experiment was tried; and, at the end of about thirty hours, she spontaneously awoke, apparently refreshed, and wholly unconscious of her protracted sleep. On the future returns of these paroxysms, which were frequent, the same plan was adopted, and she awoke, after intervals of thirty-six, forty-eight, and, on one occasion, sixty-three hours, without seeming to have suffered from want of food, or otherwise." (Vol. i, p. 372.)

It will be noticed in the above account, that it was only after the loss of some ounces of blood that the patient awoke from the lethargic sleep; and that the circumstance was dependent, apparently, upon the depletion sustained, rather than upon any pain inflicted by application of the scarificator.

The subjoined account of a case very much resembling those which have preceded, is taken from Duncan's 'Medical Commentaries,' and is related by Dr. J. Fitzpatrick of Dublin; in this instance, we have the sudden and spontaneous coming on of utter insensibility to external stimulants, with many of the outward appearances of death, the patient being in fair average health up to the moment of the attack.

"Mrs. G— P— was a full woman, about the age of thirty-five. She was the mother of several children; but, at the time of her illness, was not pregnant. She had not been so well for some time before with respect to ............ as formerly, as it had gradually diminished for some periods. But in other respects she was in perfect health.

"In this situation, as she was standing at a table, with some clothes in her hand, she suddenly, without making any sort of complaint to a maid then in the room, inclined forward, and remained in that position for some time. The maid's curiosity induced her to stir her mistress, on which she seemed to fall, or incline still more over the table, but did not, in any respect, resist the maid. This surprised the maid very much, and finding her mistress both speechless and motionless, she called for the assistance of the other servants, who endeavoured to rouse her by the vapours of hartshorn, vinegar, and the like, but to no purpose. She was then laid on a bed, a vein was opened, but not a drop of blood was discharged. In vain did they endeavour to awake her; and after spending near two hours in a variety of treatment, they laid her out for dead.

"Several hours had elapsed from the time of the attack before I saw her. Upon entering the room, I was told by a clergyman, that all was over, for that she had been dead for more than two hours. After having asked several questions with respect to the attitude in which she was first discovered, I concluded that the original attack was cataleptic . . . . . . The first thing which I thought necessary was to raise her hands, and having found them in such a state as to remain in any position in which I placed them, I had no longer the least doubt of the existence of a real catalepsia. I instantly loosed her legs, and every other structure which had been placed about her chin and breast. I removed the cold sheets, and ordered her to be wrapped in warm blankets. I directed that two girdles should be warmed, one of which was to be introduced under the shoulders and back, and the other lower down. Hot bricks were likewise applied to the soles of her feet; and her body, legs, and arms were rubbed with very strong spirits and mustard. I applied eau de luce and strong hartshorn to the nose. I introduced volatile spirits as far down the throat as I could by means of a female catheter. But all these measures, though frequently repeated, were to little purpose.

"Having tried these active remedies, together with the other agents mentioned, and not being able to discover either pulsation or breathing, excepting, as I imagined, a slight tremulous motion of the heart, my hopes were almost at an end. But I was determined to persist, in order, if possible, to relieve her . . . . . . After perseverance for an hour, she showed evident signs of being affected. In less than two hours she opened her eyes. She attempted also to speak, but could not articulate . . . . . . Her recovery went on gradually, and after it was completed, she told me that, for some time before I came, she had a knowledge of what the attendants were doing about her. But she had not, in the smallest degree, the power of using any joint in her body, of speaking a single word, or of opening her eyes. When they were accidentally opened, she could not see; so that she might be considered as completely passive." (vol. x, p. 242.)

If the identity be admitted, according to our showing, of certain mesmeric manifestations with anomalous nervous diseases, we are justified in maintaining that, so far at least, the only novelty about them consists in the mode by which they are produced; for example, that, whilst in hysteria the exciting cause of the pathological condition is mostly internal and spontaneous, in mesmerism it is artificial and designedly applied. Now, though here there may be a novel fact to some extent, there would not seem to be any incompatibility with past experience; for all analogy would suggest that what may arise from an internal or inappreciable cause, may possibly enough, under circumstances of predisposition, be brought about by outward and artificial agency. A quick pulse, palpitation of the heart, headach, diarrhœa, and other abnormal states, will commonly enough originate *ab intra;* and yet they may be induced artificially: thus, alcohol will quicken the pulse, sudden surprise will cause palpitation of the heart, headach and diarrhœa may be created in a variety of ways; and so we take it to be with the phenomena of mesmerism now under consideration: they may have their origin spontaneously, or artificially; constituting, in the latter case, no *speciality*, but merely the superinduction of certain states of the system which occasionally arise as disease.

If this view be correct, where is the insuperable difficulty in admitting the validity of *some* of the mesmeric facts—of those which are so attested, that, if they referred to another matter, they would never be doubted? The cases related by Drs. G. M. Burrows, Gooch, Cooke, and Fitzpatrick, pass unquestioned; why should not those, in their main particulars at least, of Dr. Sigmond, Mr. Ward, and Mr. Toswill, as well as many others equally well authenticated? We conceive ourselves to have disproved, or, at any rate, to have weakened, the allegation, that an *extraordinary* accumulation of testimony is required, on account of their *incredible* character. We do not doubt of the abundance of false cases, yet we are intimately persuaded that true ones are to be met with; and an indefinite amount of imposture, being but negative, can never invalidate the proof positive.

In consideration of all these circumstances, and for the whole of the above reasons, we think it is proved—or, to say the least, we think it to be made in the highest degree probable—that *there is a reality in the* SIMPLER (THE HYSTERIC) PHENOMENA OF MESMERISM; meaning hereby the *sleep*, *coma*, *altered sensibility*, *spasm*, *or temporary paralysis of muscles*, *&c.*, as provoked in certain constitutions by the magnetic manipulations; and that, at any rate, as the evidence stands, no good reason exists for rejecting them.

We have next to consider a higher and more complex order of results, stated to ensue in mesmerism; we allude to the development of SOMNAMBULISM, OR SLEEPWAKING, as it has been more suitably designated. The spontaneous origin of this phenomenon has in all ages been attested; and its occasional occurrence, as disease, is admitted by all the world. The particular inquiry to which we are now led, in our progress, would seem to be—what are the phenomena truly developed in somnambulism? After we have determined this question, we can more appropriately proceed to estimate the testimony in favour of the possibility of creating such a condition by artificial, or mesmeric, agency.

All writers on mesmerism regard the induced somnambulism as a stage of development in advance of what, for the sake of convenience, we have designated the *hysteric* phenomena; and, in this respect, a conformability is discovered between the mesmeric speculations and the statements of scientific writers on what we have regarded as the correspondent nervous derangements. "In a few instances," says Dr. Copland, "the paroxysms (of hysteria) change their character, and assume the form of *catalepsy*, *extacy*, or of *somnambulism*........... The attack may commence either as a slight or as a severe hysterical fit, and pass, in a short time, into the cataleptic or extatic state; or it may begin in the form of extacy, catalepsy, or somnambulism, and pass into the hysterical convulsion."

Before adducing cases in illustration of the somnambulistic state, we propose the subjoined formula, as defining the principal phenomena characteristic of this remarkable phasis of human existence: *A condition in which certain senses and faculties are suppressed or rendered thoroughly impassive, whilst others prevail in most unwonted exaltation; in which an individual, though asleep, feels and acts most energetically, holding an anomalous species of communication with the external world, awake to objects of attention, and most profoundly torpid to things at the time indifferent; a condition respecting which, most commonly, the patient, on awaking, retains no recollection; but, on any relapse into which, a train of thought and feeling related to, and associated with, the antecedent paroxysm will very often be developed.*

As illustrating the above singular state, and exemplifying the phenomena affirmed in the above definition, we shall supply a few cases of spontaneous somnambulism recorded by good and respectable authors.

The following instances are narrated by Gassendi, as occurring within his own experience, in the eighth book of his Natural Philosophy, sixth chapter, and third section. The cases are adduced, generally, by writers on somnambulism, including Bertrand and Prichard; and they are always referred to as genuine and authentic. They seem to furnish illustrations of exaltation of the visual faculty, compensating, as it were, for the torpor of others. Not having the work of Gassendi by us at present, although we have read the narrative in his work, we take the following account of his cases from the work of Muratori.*

"One John Ferod, well known to Gassendi, was accustomed to rise in his sleep and dress himself; he opened the doors, descended into the cellar, drew wine, or did some other such thing. Sometimes even he would apply himself to writing; and, what is very curious, although he did all this in the dark, he appeared to see as clearly as if it had been day.

"He (Gassendi) relates also that a certain Riporto, a countryman of his own, once rose in the night, and, on stilts, passed over a neighbouring torrent whilst greatly swollen; but, awaking on the opposite bank, he dared not repass it, until it was light, and the waters had subsided."

We take the following account from Dr. Prichard's 'Treatise on

* Della Forza della Fantasia Umana. Trattato di Lod. Ant. Muratori.

Insanity and other Disorders affecting the Mind.' The case exhibits, amongst other circumstances, the patient "awake to objects of attention, and torpid to things at the time indifferent," and may suggest a rational explanation of *magnetic relation*, to which we shall advert in the sequel.

"Castelli, a sleepwaker, whose case is one of the most remarkable, was a pupil of Porati, an Italian apothecary. His history has been published by Francesco Soave, a physician who personally observed him. He was found one night in the act of translating from Italian into French, and looked for words in a dictionary as usual, being asleep. His candle being extinguished, he found himself to be in the dark, groped for the candle, and went to light it again by the kitchen fire. ........ He used to leave his bed, go down to the shop, and weigh out medicines to supposed customers, to whom he talked. When any one conversed with him on a subject on which his mind was bent, he gave rational answers. He had been reading Macquer's Chemistry, and somebody altered his marks to try if he would notice it. This puzzled him, and he said 'Bel piacere di sempre togliermi i segni.'* He found his place and read aloud; but his voice growing fainter, his master told him to raise it, which he did. Yet he perceived none of the persons standing round him, and 'though he heard,' says Soave, 'any conversation which was in conformity with the train of his ideas, he heard nothing of the discourse which those persons held on other subjects.' His eyes seemed to be very sensible to objects relating to his thoughts, but appeared to have no life in them, and so fixed were they that when he read he was observed not to move his eyes but his whole head, from one side of the page to the other." (p. 441.)

Before supplying our next illustration, we shall refer to the well-known operation of the celebrated French surgeon, Cloquet, in the case of a Madame Plantin, which took place in the year 1829. The particulars are reported in the 'Bulletin de l'Académie de Médecine;' Paris, 1837, t. ii, p. 370; and they are given also in all the principal works on animal magnetism. The patient, it is stated, had laboured for several years under cancer of the breast, for which extirpation was deemed imperative. M. Chapelain, her physician, had repeatedly induced by magnetism a state of somnambulism in this person, during which common sensibility appeared to be annihilated. Under these circumstances, he proposed to M. Cloquet, who had advised the operation, that the same should take place in the artificial condition; more particularly as, in this state, her conversation and manner indicated no dread of it, whilst, in her waking moments, she repelled the idea with horror. The breast was removed accordingly; the patient not being, as in the operations of Mr. Ward and Mr. Toswill, in a state of *coma* but of *somnambulism*, for, during the whole proceeding, she conversed with the operator, indicating, however, no sensibility whatever to pain. On being awaked, she is stated to have had no remembrance of what had passed. Without discussing, at present, the validity of this account, we will *assume* its substantial accuracy; in which case, its parallel in spontaneous somnambulism may, in some respects, be recognized in the subjoined instance, related by Sauvages in the Memoirs of the Academy of Sciences for 1742, and extracted by Dr. Prichard, whose work we again place under contribution:

"On the 5th of April, 1737, visiting the hospital at ten o'clock in the morning, I found the patient in bed, which she kept on account of her debility and the pain in her head; the fit of catalepsy had just seized her, and it quitted her after five or six minutes; this was perceived by her yawning and raising herself into a sitting posture, the prelude to the following scene: She began to talk with a de-

* Pleasant, truly, to be ever removing my marks.—Rev.

gree of animation and *esprit* never observed in her except when in this state. She sometimes changed her subject, and appeared to converse with some friends whom she saw around her bed. Her discourse had relation to what she had said during her attack on the preceding day. She repeated word for word an instruction in the form of a catechism, which she had heard on the evening before, and she made pointed applications of it to persons in the house, whom she took care to designate by invented names, accompanying the whole with gestures and movements of her eyes, which *she kept open*, and alluding to the circumstances and actions of the preceding evening. Yet she was all this time in deep sleep, a fact which was strongly averred, but which I should never have ventured to declare if I had not obtained satisfactory proof by a series of experiments on the organs of sense: when she began to talk, a blow of the hand inflicted smartly on her face, a finger moved rapidly towards her eyes, a lighted candle brought so near to the organ of vision as even to burn the hair of her eyebrows, a person unseen uttering suddenly a loud cry into her ear, and making a stunning noise with a stone struck forcibly against her bedstead, brandy and a solution of ammoniacal salt placed under her eyes and introduced into her mouth, the feather of a pen, and afterwards the extremity of a finger, applied on the cornea, Spanish snuff blown into the nostrils, pricking by pins, twisting her fingers; all these means were tried without producing the least sign of feeling or perception. Soon afterwards she rose, and I expected to see her strike herself against the neighbouring beds; but she passed between them, and turned corners with the greatest exactness, avoiding chairs and other furniture that happened to be in her way, and having walked about the ward, returned between the beds without feeling her way, lay down, covered herself, and in a few minutes became again cataleptic. She afterwards awoke, as if from a deep sleep, and perceiving by the looks of those about her that she had been in her fit, she became very confused and wept all the rest of the day, not having the least idea of what had passed in her paroxysm." (p. 445.)

It is, of course, impossible to say that, in the above example, a surgical operation would have been sustained without pain; the deadened condition of common sensibility, however, as evinced by the tests employed, favours the idea that such might really have been the case.

The subjoined case, which we find in Smellie's 'Philosophy of Natural History,' was observed by Mr. Smellie himself; it displays, amongst other phenomena, a certain absence of outward sensibility which tends still further to enhance the credibility of painless operations;

"Within a mile of Edinburgh, I happened to reside some time in a farmer's house. Mr. Baird, my landlord, had a servant maid, whose name was Sarah. I had not been long there, when I learned from the family that Sarah, particularly after receiving an affront, on being angered, was accustomed to rise in her sleep, to go out, and to walk about the fields. My curiosity was excited, and I begged to be informed the first time that Sarah should rise in her sleep. A few nights afterwards, one of Mr. Baird's sons awaked me, and told me that Sarah had got out of bed. I immediately hastened to the apartment where she slept. When I arrived, Mr. and Mrs. Baird, one of their sons, and a servant maid, Sarah's companion, were present. Sarah was in the midst of them. She was slightly and carelessly clothed. Her neighbour's servant persuaded her to sit down. I took my seat by her. We began immediately to converse. She answered any questions that were put to her pretty distinctly; but she always mistook the person who spoke for some other, which gave us an opportunity of assuming any character within the circle of her acquaintance. I knew that one of the farmer's servants whose name was John Porteous, was a lover of her's; and, therefore, I addressed her in the style which I supposed John might sometimes have done. From that moment she began to scold me, upbraided me with several breaches of promise to marry her, and desired me, in the most peremptory manner, never again to speak to her upon that topic. The conversation was accordingly changed. I talked of her mistress, who was in the room, because I knew that they had occasional quarrels. Till now, I suspected that the whole was a trick, but for what purpose I could not discover. Sarah, however, abused Mrs. Baird in the harshest terms. She said, but the other day, she had been accused of stealing and drinking some bottles of ale; that her mistress was suspicious, cruel, and narrow-minded. As the mistress of the house was present, when these and other opprobrious terms were used, I began to doubt my preconceived notions of imposture; and, therefore, changed the object of my experiments and inquiries. I examined her countenance, and found that her eyes, though open, wild, and staring, were not absolutely fixed. *I took a pin and repeatedly pricked her arm; but not a muscle moved, not a symptom of pain was discoverable* . . . . . . This scene continued for more than an hour. I was perfectly convinced, notwithstanding my original suspicions, that the woman was actuated by strong and natural impulses, and not by any design to deceive. I asked if any of the attendants knew how to awaken her. A female servant replied, that she did. She immediately, to my astonishment, laid hold of Sarah's wrist, forcibly squeezed and rubbed the projecting bones, calling out, at the same time, Sarah, Sarah! By this operation Sarah awoke." (Vol. ii, p. 391.)

We shall next adduce a very interesting and well-authenticated instance of what seems to have been a sort of protracted and recurring somnambulism, during which a new character and a separate consciousness developed themselves. The following details, taken from the 'Edinburgh Phrenological Journal' for Oct. 1838, are supplied by Mr. George Combe. The communication is from Birmingham, and dated the 28th of May of that year:

"The name of the young woman is Mary Parker; she is now 16 years of age; she is of average stature for her years, slender, of the nervous temperament, with a slight admixture of the sanguine and lymphatic; she has black hair . . . . . . . . Mary Parker has had epilepsy once or twice, but during the last three years she has been subject, at intervals, to fits of a different description. When these fits are approaching, she experiences pain in her left side, in her back between the shoulders, and in the back part of her head. . . . . . . After the pain has lasted for some hours, she loses the recollection of all things and events that she knew in her natural state. She was in the house of her grandmother when she was first attacked. Her mother was sent for, but when she came, Mary did not know her, nor any person whom she had known when in her natural condition. When under the influence of the attack, she sees, hears, understands, speaks, and acts, like a person perfectly awake and in possession of the ordinary mental faculties, but there is a change in her dispositions, In her natural state, she is quiet, modest, and unabusive, showing amiable dispositions. In her new state, she is mischievous, sometimes impudent, and runs about looking out for an opportunity to do harm. She speaks disrespectfully to Mr. Jones (her medical attendant), and once threw some article at him. Her mother observed, that even when most mischievous, if a child were presented to her, she became instantly calm, she caressed it, and never injured it. These attacks lasted from a few hours to two or three days. When the disordered state is about to go off, she feels extremely weak, and lies or sits down: the fit will go off in a few minutes, and she finds herself again in her natural state, but faint and weak, and she generally asks for something to eat. She has remained well for ten or fourteen days, or sometimes more, and then another fit has commenced. In the new fit she recollects the circumstances that occurred during her previous fits, but has no knowledge of the events which happened in her natural state. After she had had several attacks, but during the period of her natural condition, her mother removed to another house. After Mary had been in it for a few days, a fit came on, and then she did not know where she was. This alternation of states has extended over three years. Of late, they have been more regular, and the fits have been more rare. She was in her natural state when I saw her; but looked pale, dull, and delicate. In her changed condition she is much more lively and energetic."

In a second communication from Mr. Combe, inserted in the same number of 'The Phrenological Journal,' and dated about a fortnight later than the preceding, the subjoined account is given:

"This day Mr. Jones having sent me notice that Mary Parker is in a fit, I accompanied him, Dr. John Conolly, and Miss —— to her mother's house. Mary was dressed, but had not come down from her bedroom, and had had no breakfast, although it was past one o'clock. She came to us when her mother requested her to do so. There was an evident change in her countenance. The muscles of her mouth and cheeks were drawn up into an expression of malice and fun, excitement was evident, something between that of hysteria and mania. The expression of the eyes was less changed than that of the lower part of the face. They did not sparkle, or glare, or look wildly, but were calm and intelligent. I asked her if she knew me. She answered 'No—how should I? I never saw you before.' Her mother asked her, if she did not recollect the gentleman with white hair, who had felt her head a few days ago, and conversed with her about her feelings. She replied, 'No, mother—I never saw that gentleman.' Dr. Conolly mentioned to her, in a very gentle and deliberate manner, several of the incidents that had occurred at my first visit, to try if he could awaken recollection, but all was in vain. It was clear that she had no consciousness whatever of having seen me before; she said to him 'I have never seen *you* before either;' which was quite correct. When asked if she knew Mr. Jones, she replied, 'Yes,' (laughing and giving him a push on the shoulder) 'I know Jones well enough.' She now wished to go up stairs to her bedroom; but her mother sat down in the stair, and completely blocked it up to prevent her. She, on observing this, moved quickly along for a step or two, and trod on her mother's toes. On her mother withdrawing her foot, and giving her a push to drive her off, she laughed knowingly, with the peculiar expression of gratified destructiveness, secretiveness, and wit.

"With a view of ascertaining to what extent she was intelligent, Mr. Jones asked her, if she could take us to the house which her mother had occupied before she came to the one in which we now were. She laughed and said she could not. I asked her 'Why she could not?' She laughed again, but made no answer. I pushed the inquiry: 'Have you forgotten the way to it?' 'No:' still laughing. 'Then why could you not take me to it?' 'Ask Jones.' Mr. Jones said, 'Tell him yourself, Mary.' She laughed archly, and then said, 'Because it is taken down;' obviously enjoying the mystery which she felt that she had cast around her former dwelling in our minds. We remained with her about twenty minutes in all, on this occasion.

"Before I left Birmingham, Mr. Jones mentioned to me that she had been twice magnetised, so as to produce a magnetic sleep. She was in her natural state, when first magnetised, and on her recovering from the sleep, she was found to be in the diseased state. She was in the changed state, the second time, and when she recovered from the magnetic sleep, she was in her natural condition."

A single instance of a case of this kind would hardly be credited. Besides this one, however, many others are recorded, which are well attested, and hitherto received by the profession as authentic. Of this kind are those cited hy Dr. Abercrombie, and Dr. Prichard. From the able treatise of the latter, we extract the following account, furnished by Dr. Dyce, of Aberdeen, to the 'Edinburgh Philosophical Transactions:'

"The patient, who was a servant girl, was first attacked by fits of somnolency during the day, which came on with a cloudiness before her eyes and a pain in her head. In these fits she talked of scenes and transactions which appeared to be as in a dream, and to follow her occupations, dressed herself and the children of the family, and laid out a table correctly for breakfast. Being taken to church during the attack, she behaved properly, evidently attended to and was affected by the preacher so as to shed tears. In the next paroxysm she gave a distinct account of the former, although during the interval she had no recollection of being at church. During the attack her eyelids were generally half shut; her eyes sometimes resembled those of a person affected with amaurosis, that is, with a dilated and insensible state of the pupil, but sometimes they were quite natural. She had a dull vacant look, but when excited knew what was said to her, though she often mistook the speaker: it was observed that she discerned objects which were but faintly illuminated. The paroxysms generally continued about an hour, but she could be roused out of them; and then she yawned and stretched herself, like a person awaking out of sleep. At one time she read distinctly a portion of a book that was presented to her, and she sang much better than in the waking state. After six months this affection ceased on the appearance of the catamenia."

There might be cited other cases of alternating consciousness, or *dypsychia*, as it has been termed by Dr. Prichard, spontaneous and artificial, but those already given are sufficient for the purpose of exemplifying the actual characters of that modification of *hysteria* (the term being employed in its ordinary and widest acceptation) denominated *somnambulism.* And although it is likely enough that some little exaggeration or colouring may pervade the above narratives, the evidence in support of the main phenomena, as arising spontaneously, is undoubted, and undergoes no question; it satisfies men like Dr. Abercrombie and Dr. Prichard, of high and established reputation, accustomed to investigate circumstances and to balance testimony relating to such things; and, however curious and extraordinary the cases may appear, they are, when related by honest and able men, whose authority would be received on other corresponding subjects, admitted to be genuine. Now, if states of the system so abnormal may arise from inward and inappreciable causes, where is the reason for doubting that, under some circumstances, they may be provoked by the mesmeric processes? The *onus probandi* of the contrary lies with those who maintain that it cannot be so. In the impossibility of personally examining cases, or in the absence of any valid testimony to the fact, the power of inducing somnambulism by artificial means might very fairly be *questioned;* but knowing so little as we do of the causes of this affection, even when it arises spontaneously, how shall we make out, *a priori*, that mesmerism, under no circumstances, can lead to its production? It is purely an affair of fact, to be determined by observation; and where from any cause this may be impracticable, its credibility must be regulated by the evidence. We have ourselves witnessed instances of somnambulism, induced by magnetic manipulations, and have not doubted of their reality. We have observed individuals who had submitted to the practice, to fall down, as if in deep sleep, and manifest no signs of outward sensibility, and then to arise out of this state into one closely resembling that in the instances we have adduced of spontaneous somnambulism, evincing, most commonly, torpor of the senses, unless there was some special excitant to a particular object; the patient holding conversation sometimes with the mesmeriser only, at others with bystanders indifferently; and doing a number of acts, and exhibiting various sensibilities, which indicated *some* though *anomalous* communication with the external world. In one well marked instance, where the mental activity was very considerable, we elevated the eyelids, noticed a staring vacant look, the pupils widely dilated, and utterly insensible and incontractile on the closest approach of the flame of a candle. Great exaltation of sensibility, and great depression of the same, we have remarked in these cases; and partial torpor, compensated by elevated susceptibility elsewhere, we have also witnessed.

If, from any circumstances, actual cases cannot be witnessed, testimony alone must supply the materials for a decision concerning their reality; and, here, we do not see that the evidence to prove mesmeric somnambulism need be higher or stronger than that which is deemed requisite to demonstrate its spontaneous analogue. Any one who shall take the trouble to examine some one of the leading works on animal magnetism will readily perceive that, all reasonable allowance being made for exaggeration in some cases, and misrepresentation in others, the attestations are as numerous, and some of them apparently as decisive, and from as well-informed and intelligent witnesses, in favour of *provoked* as of *natural* somnambulism.

Exactly on the same grounds, then, that guided us to a conclusion in dealing with the simpler, *hysteric* results of mesmerism, do we admit the reality of the somnambulistic phenomena within the limits of the definition given in a foregoing page, and maintain that, in the actual state of the question, they cannot reasonably be rejected; that they are clearly within the boundaries of ordinary medical science, presenting no characteristics at variance with past experience; and that, consequently, there is nothing in them by which we ought either to be startled or confounded.

We have said *within the limits of the definition given in a foregoing page*, because there are characteristics recorded of somnambulism, which certainly our formula will not comprise; we allude to what have been termed the phenomena of *lucidity;* by which is understood the display of certain qualities and capabilities said to arise in somnambulism in its highest stage of development.

The statements we have now to investigate are entirely distinct, both in nature and in credibility, from any that we have hitherto examined. So far from harmonising with received laws or principles, they, in many respects, oppose themselves to all ordinary notions concerning either the probable, or the possible; the evidence, therefore, required to prove them, must, in extent and completeness, far transcend what we might deem adequate to assure us of such facts as those with which we have as yet been engaged. Indeed, with respect to some of them, it may be a reasonable question, whether any conceivable amount of testimony should lead us to admit their possibility even,—excepting on principles that would justify the recognition of miraculous interposition. For, we shall presently show that, according to the more advanced disciples of the mesmeric school, a lucid somnambulist is endowed with the faculty of enunciating information upon almost any conceivable subject, however unlearned or ignorant he may be in the waking state; he can tell what is going on about him or at a distance without using his own eyes or those of other people; the past is known and the future foreseen; his own diseases and those of others, near and afar off, are diagnosticated and successfully prescribed for; in a word, the lucid sleepwaker is represented as knowing almost everything; and all this, not in the ordinary way by observation and reflection, but by a sort of mystic intuition. Thus, Mr. Colquhoun states, in his 'Isis Revelata,' that "the eye of the mind, the internal power of vision, is wonderfully strengthened and enlarged, and seems unconfined within the narrow limits of space and time; we do not see objects in a merely superficial manner—we penetrate beyond external nature." M. Teste, from whom we shall next quote, is a French physician, and a member of several learned societies; his 'Manual' has recently received an English dress, and is, by permission, dedicated to Dr. Elliotson. From these circumstances we infer that M. Teste is an admitted authority; and, on this account, we shall draw somewhat largely upon him for particulars of the phenomena of lucidity. We find it stated, then, by this author, that the condition in question is often characterized by "vision without the aid of the eyes—intuition—interior prevision—exterior prevision—penetration of thought—transposition of the senses—the instinct of remedies." With respect to the faculty of seeing without the customary organs, he affirms that there is no magnetiser who has not witnessed its manifestation twenty times. *Intuition*, we learn from the same writer, initiates, suddenly, the intelligence of the subject in whom it occurs, into the sublimest mysteries of his intimate nature.

"One could never imagine with what tact, justice, and precision somnambulists ascertain what passes within them. They assist, literally, in the accomplishment of all their organic functions: they discover therein the most imperceptible disorder, the most fugitive alteration. There are no ailments so slight or so latent, those even which, in the earliest periods of their existence, not only furnish no external symptom, but yet manifest themselves by no species of internal suffering; there are no ailments, I say, which escape the investigation of the somnambulist. Then of everything there is formed an idea which is exact, rigorous, and mathematical. He would say, for example, how many spoonfuls of blood there are in the heart; he knows, to a nicety, how much bread would be required to satisfy his appetite at the moment; how many drops of water to appease his thirst; and all his estimates are of an incomprehensible exactness. Time, space, all kinds of forces, the resistance and weight of objects, his thought, or rather his instinct, measures, calculates, appreciates all these things in the twinkling of an eye. A woman in somnambulism hath the consciousness of her pregnancy from the first hour of conception; she feels whether or not she be in the disposition to conceive; finally, she will not be pregnant eight days before she will designate the sex of her child, *and never be deceived*." (Teste, p. 115.)

By the power of *interior prevision*, "somnambulists," says M. Teste, "announce, by a sort of prescience proper to themselves, all the modifications destined to happen in their organism." With respect to *exterior prevision*, the same authority informs us, that "some subjects possess the incomprehensible faculty of predicting certain events, with which their own existence shall be found to have concern, but the cause of which, obviously foreign to their own economy, could have no explicable sort of relation with it." *Penetration of thought* is the singular faculty with which "certain extatics and a small number of somnambulists are endowed, of penetrating the thoughts of persons who are about them, before the thoughts have been in any way expressed." *Transposition of the senses* is said to occur in subjects who see, feel, taste, and hear by the stomach or by the ends of the fingers. The *instinct of remedies*

enables the somnambulist to prescribe, with high probability of success, for the ailments of persons with whom he is placed in relation, and the defects of whose organism, actual or in anticipation, he readily discovers.

Dr. Passavant, of Frankfort-on-the-Main, has long been a writer of standing among the mesmerists; and from him we shall take a brief account, which is given in the work named at the head of this article, of a lucid somnambulist who exhibited the phenomena in question:

"She attained this condition of vision first by magnetism, later by the mere will, and in this state she saw the persons and things whereupon her will rested. Frequently she described, with perfect accuracy, the bodily and spiritual condition of men whom she had never seen, and administered curative agents, such even as in her waking state were totally unknown to her; or she ascertained that the means of cure did not exist, as in organic defects for example. It depended upon her will to remember or not, on awaking, these interior observations." (p. 93.)

Dr. Caldwell, a well known American physician, who has written on hygiene and some other medico-philosophical subjects, has published a pamphlet on mesmerism, in the preface to which we find the following statement:

"For one person completely to identify another with himself—sense with sense—sentiment with sentiment—thought with thought—movement with movement—will with will—and I was near saying existence with existence—and to gain over him so entire a control, as to be able to transport him, in his whole mind and being, over mountains, seas, and oceans, into distant lands, and disclose to him there the objects and scenes which actually exist—of which he was utterly ignorant before, and becomes alike ignorant again, when restored to his usual condition of existence; and, higher and grander still, to waft him at pleasure through space to any or all of the heavenly bodies, of which we have any knowledge, and converse with him about them—such deeds as these may well be called amazing. Yet are they as easy, certain, and speedy of performance, as many of the most common transactions of life." (p. xxi.)

Can such things be, and overcome us like a summer's cloud, without our special wonder?

Quis latet hic Superûm? Quod numen ab æthere pressum

Dignatur cæcas inclusum habitare cavernas?

Quis terram cœli patitur Deus, omnia cursûs

Æterni secreta tenens, mundique futuri

Conscius, ac populis sese proferre paratus?

The above statements, from accredited writers on animal magnetism, respecting the characteristics of *lucidity*, furnish a fair specimen of the averments generally to be found in mesmeric literature; and we can assure the reader, that in our selection, we have been influenced by no wish or intention to obtain an appropriate butt whereat to aim the shafts of ridicule. We have a very different design. However tempted to make merry with, or to scorn, the assertions concerning mesmeric lucidity, our resolution has been to realize the scheme declared at setting out, to examine the entire subject calmly, and without prepossession; and to do this in conformity with the method of investigation already enunciated.

The phenomena in question may be classified under two heads: first, those which, if supported by personal observation carefully and repeatedly made, and backed by corroborative testimony of the highest kind, might be admitted as possible; secondly, those which, as natural facts, could hardly be received upon evidence even the most overwhelming. We leave the reader to arrange the instances according to his own judgment; and shall at once proceed to the more important subject of examining them in detail. We may first observe, however, that, with many other unbiassed inquirers, we have anxiously sought for these wonders, when opportunity has arisen, but have never been enabled to find them; our only resource, therefore, is to discuss the value of the evidence in attestation of their existence, and to see if it correspond with the just requirements of a sound philosophy.

Little discussion, we apprehend, will be demanded to show that the higher phenomena, so called, of animal magnetism, as facts arising in the order of nature, are at utter variance with past observation. We know that isolated circumstances have at various times been recorded, apparently in opposition to the present assertion. We have read of the extravagances and the reveries of the Montanists of old, of the thaumaturgic doings of the Priest Gassner, (dismissed as an impostor by his Bishop,) of the convulsionnaires of St. Medard, of the prophets of Cevennes; and we are aware that there are other narratives of a like kind. A belief in the reality of the wonders mixed up with these histories was, however, never general, nor did it endure to any extent, beyond the period in which the excitement, occasioned by the actual facts, prevailed. It has only been of late, since the rise of *lucid mesmerism*, that the marvellous portions of these published accounts have been revived, apparently with the view of facilitating its reception. Certainly, all *generally* admitted experience has shown, that sight is dependent upon the eye, and that, without the mediation of that organ, vision is impossible; further, that for information to be enunciated, it must have been received through the senses; and that the power of "prevision" or of *seeing* into futurity, as distinct from *inferring* probable occurrences, is no *human* faculty. The miraculous events, on which rest the foundations of religious belief, cannot rightly be adduced to the contrary; these things appertain to a higher philosophy; they have ever been admitted and received as *out of the order of nature*, and, on that account, as *miracles*. The assumed facts of lucidity, however, are presented to us as *natural*, and they must so be discussed. In this point of view, we conceive it to be beyond dispute, that they seemingly contradict, or oppose themselves to every well-authenticated record of the past, and contravene at the same time the universal consent of mankind. Yet does this, by itself, furnish no good ground for their absolute rejection. The Emperor of Japan had, to himself at least, a similar reason for discrediting the Dutch merchants, when they affirmed that certain rivers of Europe became at times sufficiently solid to sustain the pressure of human footsteps, and even the weight of horses and heavy vehicles. Yet, in this case, it need hardly be said, an investigation of the whole affair would have invalidated the Emperor's conclnsion, notwithstanding its accordance with past experience and anterior notions.

Is it so, however, with the phenomena of lucidity? And, in the other case, ought any amount of *ordinary* evidence to be deemed adequate to the proof of their reality? If it should even be made out that the testimony in favour of *lucid* somnambulism is as strong as that in support of *simple* somnambulism, are we as much bound, philosophically, to admit the one as the other? We contend for the negative. Evidence, as before maintained, must ever correspond in strength with the extraordinary and anomalous character of the thing to be proved; and thus, while attestation, of ordinary value and extent, may yield proof with respect to matters which harmonize with ascertained facts, none that is not of the very highest order can, or ought to be deemed decisive with regard to statements of a contrary kind. But we think it may safely be asserted that the testimony in support of the glaringly improbable facts in question is even *less* strong than that which is considered requisite to establish an *ordinary* philosophical proposition. It is neither complete, nor free from suspicion; and, when its fallacy cannot be demonstrated, it is yet so susceptible of being explained away, as to lose its conclusiveness. Evidence to be *complete* must not only come from valid witnesses individually considered, but the statements must be decisive and coincident amongst themselves. If, however, we consult the generality of the recorded examples of lucidity we shall notice that almost invariably there are no careful and precise details, showing that suitable precautions have been taken to remove the sources of fallacy; and that the cases themselves, for the most part, are, confessedly, a compound of hits and misses.

Moreover, the absence of uniformity in the results, obtained by observers of lucid phenomena, will not fail to strike an inquiring mind. One magnetiser reports one class, as constantly to be met with; another, never having seen anything to correspond, has, however, encountered other manifestations at every step. Thus, M. Teste considers himself so familiar with extra-ocular vision, that he ventures to affirm, there is no magnetiser who has not seen it at least twenty times; Dr. Elliotson says that, after six years' constant search, it is only very lately that he has witnessed one or two such cases. M. Petetin, in a work on 'Animal Electricity,' states that he had experimented upon a number of cataleptic women who, quite deaf to sounds in the common way, heard plainly when he whispered to them near the epigastrium; this, a somewhat frequent phenomenon in M. Petetin's experience, is but rarely heard of in that of his magnetic confrères. In the Rev. Mr. Townshend's work, almost every recorded case of somnambulism exhibits community of taste, on the part of the mesmeriser and mesmerised; some eighteen months ago, we know that Dr. Elliotson stated to an unexceptionable witness that, with all his accumulated experience, he had then never witnessed the phenomenon. Deleuze and most other writers on magnetism dwell constantly on the influence of the magnetiser's *will* on the moral and physical condition of the magnetised; others again, of equal experience, affirm that they never can recognise any influence except from *material* gesture. This particular discrepancy is most remarkable, and damaging in the extreme to mesmeric testimony. It is worthy of notice that the points of difference in all these matters generally mirror the mental differences of the observers. The avowed materialist, like Dr. Elliotson, rarely encounters manifestations so sublime and spiritual as those attested by Townshend or Deleuze. Does not the inference suggest itself? When we have proposed this difficulty to professed magnetisers, we have been told that the diversity in result came from the circumstance of different observers being on the look out for different phenomena. In this explanation, we believe the truth to be included; we suspect, however, that earnestness in the look out may have conduced more to excitement of the fancy than to accuracy of observation; and that the variations we have mentioned are inherent, rather in the minds of the observers than in the objects observed.

Nor yet is the evidence, incomplete and contradictory as it is, free from suspicion on other grounds. The phenomena are nearly always reported to have arisen in subjects upon whom experiments had been made for a long previous period, and who are considered to have been gradually *wrought up* to the state in question; and the results obtained pretty generally correspond with the favorite hypothesis of the particular mesmerist. When it is considered how thoroughly characteristic of hysteria, cunning, and clever deception are ascertained to be, ought not such traits at least to be suspected in all unwonted mesmero-hysteric instances? If our own inference respecting the analogy or identity of mesmerism with hysteria should be disputed, the argument is but little affected, since it is fully allowed, by most mesmerists, that magnetic somnambulists are ever deceptive, vain, and fond of effect. For our parts, we have little doubt that, both in spontaneous and artificially-induced hysteria and somnambulism, there is frequently a *superadded* deceit—a moral symptom of the disease itself, and not an indication that *all* is imposture. In researches into matters of this kind, we contend, not only that it is *not* "cruel and ungenerous to be ever suspecting young, innocent females, of excellent character and disposition," as Dr. Elliotson and others too confidingly maintain, but that it is just and philosophical to do so, even under circumstances where the motive can neither be understood nor imagined. When anything anomalous seems to develope itself in hysterical persons, suspicion of deception, we reiterate, should *always* be entertained. Were we to enter fully into the proof of this position, we should consume too much space; and, indeed, addressing, as we are, medical men, it is not at all necessary. Let it, however, be constantly remembered, that hysterical subjects are nearly always the persons reported as *lucid.*

Positive circumstances, moreover, occur from time to time, not merely throwing over the best-authenticated cases the greatest distrust, but proving moreover their utter falseness. These, in the distance, or in the absence of *critical* examination, have often seemed decisive and circumstantial enough; when, on coming nearer, or when looked into with a cool and unprepossessed spirit, the reality of the marvel has become dissipated most lamentably. These wonders are too generally like the *Fata Morgana:* afar off, all is beautiful and distinctly defined; on approach, the very outlines have vanished, and are nowhere to be found!

The proceedings of the French Academy of Medicine, in their inquiries relative to animal magnetism, exhibit abundant illustration of the statement just made. It may be known to most of our readers that this body has, at several periods, instituted commissions, charged with the examination of evidence bearing upon this subject. The reports which have been issued at various times, recount numerous cases of alleged lucidity, and at the same time display their most inconclusive character. We shall here content ourselves with noticing a few of these: but they are the choicest specimens adduced by the magnetisers.

The alleged phenomena of lucidity being so very extensive, and the statements with respect to them being so vague in relation to the qualifying circumstances, the Academy consented to make the faculty of seeing without eyes—clairvoyance—the touchstone of experiment regarding the reality of the state in question. Moreover, on the 5th September 1837, M. Burdin, one of its members, offered a prize of 3000 francs to any person who should display the faculty, by reading through an opaque substance. The money was deposited with a notary, not to be withdrawn until after the question should be decided. The parties appointed to act as judges in this matter were impartially chosen, and consisted of MM. Dubois, Double, Chomel, Husson, Louis, Gerardin, and Moreau. As the proposal was extensively published, it was reasonable to suppose that the best cases would present themselves, not only for the pecuniary gain's sake, but on account of the opportunity afforded of proclaiming to the world so public and so distinguished an attestation of the fact, if it had any existence. Indeed, the applications were many, and from great distances. Written and seemingly-authenticated accounts were, in most instances, sent beforehand to the Academy; on proceeding to the trial, however, the cases always broke down. Any one who shall peruse the account of these claims furnished in the *Academic History*, will perceive that every imaginable concession was made to the competitors, compatible with the object in view—that of testing the reality of the power. The results, however, never led to any positive conclusion; vision through opaque bodies, or without the eyes, could never be demonstrated. The prize had originally been proposed as open but for two years; an additional year was conceded; it was all in vain, and the prize was finally withdrawn. We have never heard of any serious objection to the justice or the impartiality of the umpires on the occasion.

The three following illustrative cases are abridged from the work of MM. Burdin and Dubois:

In Oct. 1837, a physician of Provence, M. Houblier, addressed a letter to the Academy setting forth that, for the space of a year, he had been constantly engaged in magnetic experiments, and had succeeded in developing nearly all the phenomena mentioned by authors; and that he was anxious to compete for the pecuniary prize, but that he should require some little time for the due preparation of his somnambulist previously to a demonstration so important. It was, however, nearly two years, before M. Houblier became sufficiently confident—not to present his subject, Mlle. Emélie, to the commissioners but—to forward to them an account in detail, of certain experiments indicating her ability to read a book, when placed behind the back. No time, however, was specified by him for the required journey to Paris. Another year had almost elapsed, before M. Houblier, was again heard from; when a second detail of successful experiments was sent to the Academy, but still there was a shrinking from the actual trial; and M. Houblier preferred a request for a further extension of time; this, after a lapse of nearly three years of preparation, was very properly regarded as mere trifling, and the demand received no attention. However, in the month of Sept. 1840, the somnambulist was at length sent to Paris, and placed under the care of M. Frappart, a friend of M. Houblier, and a cultivator also of animal magnetism. The subsequent history of Mlle. Emélie was published by this gentleman in the *Gazette des Hopitaux*, of the 31st Oct. 1840. From this account, it appears that shortly after her arrival, and preparatory to her presentation before the commissioners, M. Frappart, fully confiding in her powers, commenced himself to experiment upon her ability to read with the occipital region. The precautions which were taken to obviate fallacy, however, constantly led to complete failure; a succession of excuses in explanation were made by the somnambulist, and admitted;—she was not well; the fatigue of the journey, the change of diet had disturbed her, &c. For a while, all these averments were regarded by M. Frappart as likely enough to be just. After reiterated attempts, all abortive, Mademoiselle at length adduces the plea of sickness, and "fears she has taken a fruitless journey, and is perplexed moreover by the number of books in the library, *readinq one for the other.*" Her temporary guardian now begins to suspect her good faith; but, for the purposes of discovery, affects to sympathise with her; suggesting that it might be well to have only one book in the room, which she could study whilst sleeping, and that, to aid her, he should leave the room until the lucidity was complete. M. Frappart observes that the snare laid was so obvious, that the silliest mouse should hardly have overlooked it; it appeared, however, that Mlle. Emélie suspected nothing, but readily assented to the proposal. "I set my trap," continues M. Frappart; "Mlle. Emélie shall sleep there; here shall be the book, the doors shall be closed, and through this little hole I shall have my eye fixed upon the book." The somnambulist is summoned, she lies down, and is put to sleep by the agency of a magnetised ring, without any interposition on the part of M. Frappart. A book, the only one in the room, is placed upon a chair, a few steps behind Mademoiselle; he leaves the room, is away from the door for about five minutes whilst hastily breakfasting, and then resorts to his hiding-place, where he remains for nearly half an hour; after this lapse of time, the somnambulist, according to previous arrangement, rings the bell, as an announcement that her clairvoyance is complete. M. Frappart enters, never having seen the book removed; and yet, to his surprise, several words were distinctly made out. If there were trickery here, he reasons, the book must have been examined in the brief period he was away from the post of observation. On the following day, the experiment was repeated with greater care; and, on leaving the room, he commenced at once to watch through the key-hole. In about ten minutes, the book was seized by Mademoiselle, carefully examined here and there, and then returned to its place. The bell rings, and M. Frappart enters. Emélie read, as before, some few words, obviously to the extent of her previous preparation by searching the book. The discovery just made is not announced, but steps are taken for demonstrating the imposition to several other persons interested in the inquiry, and especially to M. Houblier himself, who, at once on receipt of a letter from his friend, sets off for Paris. The fact was exhibited to all by a repetition of the same manœuvre, and the young woman was, of course, never presented to the academic commissioners. Yet she had, for a long period in Provence, imposed upon many influential and respectable persons, besides her immediate protector, M. Houblier, who, on this exposure, acknowledged with grief and shame that he had, for four years, been the dupe of this artful person (une maitresse femme.) Had a book been written by some Provençal magnetist, just prior to the Parisian excursion, undoubtedly the case in question would have been recorded as supplying evidence of lucidity that was at once circumstantial, decisive, and irrefutable.

On the 10th of Oct. 1837, M. Pigeaire, of Montpellier, addressed a letter to the commissioners, recounting the history of his daughter's lucidity, and concluding by accepting the proposition of M. Burdin. From the details afforded in the communication, it appeard that Mlle. Pigeaire, a little girl 11 years of age, had recognized, in magnetic somnambulism, the arrival of a visitor when only at the street door; that she was, in this state, able to name the contents of a snuffbox when closed, and to see the interior of another person's body; in a word, that she was a complete and genuine clairvoyant. The counter-proposition of M. Pigeaire, in offering to compete for the prize, was in the following terms: "My magnetic somnambulist shall say to M. Burdin: Render me temporarily blind, be confident that the least light cannot reach my eyes. *If, in this state, I do with my fingers what you shall do with your eyes, that is to say, if I read with my fingers, which then become my visual organs, a plate of glass being applied on the printed characters, or hand-writing, which you shall give me to read; if, by these new organs, I transmit to my brain the tenour of the writing which you shall have given me, and if I express it to you without any mistake, I shall doubtless have fulfilled the conditions of your programme; but you must recollect that, in order to fulfil this condition, all the properties of the object which my fingers look at must be accessible to light*; *otherwise, how should I see them? How should my fingers convey to my brain the colour and form? In one word, put me in the conditions for reading with my fingers as you read with your eyes.*" Although this proposition scarcely harmonised with some of the conditions laid down by M. Burdin, it was yet acceded to, because the concession "render me temporarily blind" seemed to permit the employment of all reasonable means for securing the exclusion of light from the eye; and, in the month of April 1838, M. Pigeaire, with all his family, arrived in Paris. Although, in the previous correspondence, he had spoken of the delicacy of the affair, seeing that the somnambulist was his own daughter, he does not proceed quietly and unostentatiously to present her to the commissioners, but in the first instance makes her the subject of public exhibitions, gathering together physicians, newspaper editors, distinguished literary persons, peers even, and other people of rank. Signatures attesting the genuine character of the case were obtained from many of these parties. "However," says the Academic History, "the commissioners quietly waited till it should please M. Pigeaire to show them his somnambulist; they were men severe and exact, not at all anxious to mingle themselves with peers of France and deputies. It was of no importance to them whether George Sand* gave or refused her signature." Two months after arriving in Paris, the commissioners received a communication from M. Pigeaire, setting forth that, in the ensuing trial, his daughter's eyes must be bandaged with an apparatus of his own, constructed of black velvet, and that this must be adjusted by himself. This gave rise to much discussion, it being contended by the commissioners that, here there was a manifest departure from his own "render me momentarily blind," terms which implied that the mode of shutting out the light should be fixed upon and be carried out by some one or more of themselves; that, be-

* A *nom de guerre* assumed by Madame Dudevant.—Rev.

sides, the velvet bandage could easily be disarranged, so that, beneath its inferior border, vision to some extent could take place in the ordinary way. Notwithstanding all this, M. Pigeaire would listen to no other plan; every proposal, interfering with his own method of applying his own bandage, was, for some reason or another, constantly rejected; at length, after many conferences the commissioners surrendered, on the single condition that, while the objects to be descried "should be placed at the *distance* required by M. Pigeaire, and in a perfect light, the *direction* should be in exact opposition to the eyes, so that if some slight separation of the bandage, at its inferior margin, should occur, nothing could in that way be seen." Even this simple provision against fallacy was disallowed; the object that was to be distinguished by the somnambulist must be held by herself, and in the position that she wished! It is almost needless to say that, hereupon, negotiations ceased.

It is unnecessary to exhibit, by any argument, the worthlessness of the evidence presented by this case; the palpable shrinking from the proposed competition, after all that had preceded, furnishes materials for determining its true character far more decisive, in our estimation, than any recital of wonders that had been displayed at Montpellier, and at the public exhibitions in Paris, even though supported by the written testimony of peers, deputies, and George Sand.

Now, if clairvoyance had been a reality, would not the Burdin proposal, according to every law of probability, have led to a demonstration of the same—the faculty whose manifestation, according to M. Teste, every experienced magnetiser has seen at least twenty times? Before we close our notice of the Academic prize, we shall furnish some particulars of one other claim that was made, and that by no less a personage than M. Teste himself.

It was in the third and last year of those in which the prize was open to competition, on the 1st September 1840, that M. Teste addressed to the president of the Academy the following letter:

"Mr. President,—Having succeeded in producing an experiment which appears to me calculated to lead to a sure judgment, if not concerning the question of animal magnetism, at least upon that which relates to the phenomena of seeing through opaque bodies, I consider it a duty to propose to the academy the examination of this experiment.

"I refer to one *and even to two* somnambulists reading through the walls of a box of pasteboard and even of wood, the only condition indispensable to the success of the experiment being that the direction of the letters inclosed in the box should be previously designated.

"I presume to hope, Mr. President, that this letter will be read at the first meeting of the Academy, and that none of your honorable colleagues will refuse their consent to the examination of an important fact, and one the consequences of which may be of immense importance to science and *humanity*." "Signed Teste."

This proposal was at once accepted; and it was conceded that the box should be constructed of any substance M. Teste should wish, excepting glass that was decidedly transparent. Accordingly, on the 5th of September, the commissioners, including MM. Husson, Louis, Chomel, Gerardin, Dubois, and Double, assembled by appointment at the house of M. Teste; and they had been requested to bring with them a pasteboard box, inclosing some lines in printed character.

The subject, a young girl, was introduced into the room, and magnetised by M. Teste; very soon she was declared to be in somnambulism; and the box inclosing the printed paper was handed to her, the direction of the lines and the letters being indicated according to the previous conditions. A little after, M. Teste asked the alleged somnambulist if she would be able to read in the interior of the box; she answered in the affirmative. He asked her in what time she would be able to read; she answered: in ten minutes; and all that with an assurance and confidence truly remarkable. We shall supply the sequel of this narrative by a verbatim extract from the 'Academic History:'

"However, the somnambulist looked at the box, moved it, and turned it within her hands. In her movements she tore one of the bands that served to seal the box. This was openly remarked upon, and the thing was not pushed any further. The embarrassment of the somnambulist appeared to increase; she exerted herself vainly in efforts, in appearance at least, very fatiguing. The length of the lines (being verse,) did not fill the entire length of the box; there was a considerable space of white paper, and to this space especially were the attention and the fingers of the somnambulist directed, as if striving to spell where there were no letters. She had announced her ability to read in ten minutes; half an hour, an hour even, had passed away thus. The magnetiser asked the somnambulist how many lines there were in the box. She said there were two; he pressed her to read; she announced that she saw the word *nous*, and later the word *sommes; nous sommes.* Finally, the somnambulist having declared that she could read no more, the box was taken from her hands; the magnetiser caused the magnetic sleep to cease, and the somnambulist quitted the room.

"The box was opened shortly afterwards in M. Teste's presence; the fragment of printed paper which it inclosed contained the six following lines. . . . .

Encore un mot, Romains, tout est mûr pour la gloire,
Ma dernière parole est un cri de victoire;
Nos succès fussent-ils différents ou douteux,
S'arrêter est fatal, reculer est honteux.
Choisissez; Rome libre ou la patrie esclave,
La mort, effroi du lâche, est la palme du brave.

It is then seen that the box did not contain two merely, but six lines; and in these six, there was neither *nous* nor *sommes.* The experiment failed completely." (p. 629.)

We shall further exemplify the feeble character of the evidence to prove clairvoyance, in recounting certain details of two more recent cases which did, for a while, seem to furnish testimony the most conclusive that lucidity, after all, was a fact. We think it right to state our own opinion that the instances in question, up to a certain period, supplied documentary proof equalling at any rate, if not surpassing, the very best in support of other such cases. With the outlines of the first illustration, many of our readers are probably familiar.

The arrival of Alexis in this country in the summer of 1844 had been preceded by his fame. For some months previously, the newspapers and journals had related the most wonderful stories of this youth's extraordinary powers. There was nothing, in the way of lucidity, he had not done and could not do. He could describe the contents of any room of any house never before seen or heard of; he had divined the contents of sealed packets, and thick wooden boxes; he had revealed to anxious authors the unknown lodgement of important papers; and a thousand things besides equally marvellous. He was considered, we believe, by the leading mesmerists of this country, to constitute their fairest specimen. On his arrival, one of the most influential of the metropolitan journals supplies copious details attesting the lucidity of Alexis. Here, at last, thought we, doubt and uncertainty come to an end ; here is what we have been so long anxious to see, a case of downright, unequivocal clairvoyance, at least. Accounts, however, published by other papers, exhibited the lucidity in a manner dubious and equivocal enough. The same hits and misses, the same half-right and half-wrong, the same extenuation of failure, that we so often encounter on approaching these lucid somnambulists. We take up the 'Zoist,' the accredited organ of the mesmerists, and expect there at least to have some true and particular account of the successes such as they are described and vaunted in the systematic books. What do we read? Statements exhibiting, certainly, amazing power and accuracy, as to some matters ; but, even here, much hesitation and great blunders as to many others.

We presume that it was felt by the parties who had hopefully anticipated the production of Alexis in London, that the demonstrations did not realize previous expectations: the following is the explanation of the failures, furnished in the 'Zoist:'

"The power was on Alexis at times only; coming in gushes or flashes, as forced states of the living body do ; pain, convulsions, flashes of light, noise in the ears, emotion, and even the inspirations of genius. This should be carefully remembered. The state is a forced state ; and though, if strong, it is more uniform ; if weak, it will flicker. It must also be remembered, that he unfortunately thinks aloud; names each appearance and thought as it presents itself to him, and therefore seems to guess; whereas he is like a man reading an ill-written letter, or looking at very distant objects, who fancies one word or object and then another, till at last he is satisfied what the real one is. He, therefore, is often apparently in great error when he first speaks; and, though nothing be said by others, he goes on correcting himself. It would be well if clairvoyants said nothing and had nothing said to them, till they felt themselves certain."

We are willing to allow that this quotation would yield a very fair reason for the seeming guesses of Alexis, if the question were of some individual illustration of a general fact already established ; in the present state of matters, however, the explanation will not satisfy the justly sceptical mind ; for let the whole account of this instance of lucidity, with its qualifications, be compared with the announcement, in mesmeric systems or manuals, of positive, unmistakeable indications and manifestations of this state, and it will be seen how immensely the English displays of Alexis come short: and, where *shall* we look for the lucidity of the books, if not in a subject of European celebrity?

We shall not pursue the story further in detail : most of our readers very likely are aware of certain experiments in this case conducted by the Editor of this Journal, and of the very unsatisfactory results, to say the least of them. By all the persons who witnessed the last investigations at the house of Dr. Forbes, we believe, with the single exception of M. Marcillet and one other avowed mesmerist, they were considered to have established the proof of downright imposture ; in our extreme caution, we do not go so far as this, as the results were all negative, but we do maintain that they were adequate to show in the most decided manner that, in the person of Alexis on the occasions in question, *lucidity*, to say the very least, was unproved.

In the last number of the Zoist, that for January of the present year,

Dr. Elliotson has collected a variety of attestations to show that Alexis was truly and faithfully all that, before his arrival in London, he had been represented. And, indeed, on a careless examination and afar off, all does appear circumstantial and definitive enough; but the critical eye sees defective evidence and room for delusion or collusion in almost every one of the recorded cases. Why is it that the marvellous ever vanishes on approach and under critical investigation? The following would seem to be the reason afforded in Dr. Elliotson's paper: "M. Marcillet immediately predicted that Alexis, with such a person (an incredulous one) at hand, would not succeed. Such his invariable experience showed, although he allowed he could not account for it." (p. 491.) The same gentleman excused the failures at the house of Dr. Forbes, by the *atmosphere of incredulity* ("l'atmosphère d'incrédulité") which he said existed around him, emanating from the persons of the sceptical doctors present,—although these unconscious poisoners of the air, all (with one exception) sat in solemn silence at a considerable distance from Alexis.

The reason assigned in Dr. Elliotson's paper is, doubtless, a very convenient one for the mesmerisers, but surely it is not such as can for a moment weigh with a philosophical mind, when there is question respecting the very existence of such a power as clairvoyance.

As a further illustration of the way in which cases of this kind come to nothing as evidence in proof, when made objects of careful and critical investigation, we shall adduce another little history, which we gather partly from certain numbers of the 'Manchester Guardian,' issued last April and May, and partly from information supplied to us by a gentleman, in the accuracy of whose information we have every confidence.

Mr. Hewes, a gentleman of property residing in Greenwich, educated for, though not practising, the medical profession, having, during a temporary residence in Germany, been witness to the wonders of animal magnetism, commenced, on his return home, to devote himself with energy and assiduity to the practice of the art. Amongst others upon whom he had exercised his powers was a young man of about 18 years of age, called "Jack;" the effect being stated to be the production in him of genuine and unquestionable clairvoyance. Hereupon, Mr. Hewes resolved to exhibit this marvellous phenomenon in some of the principal towns of the kingdom, commencing with Manchester as his native town, and the place from which he draws a large proportion of his income.

Accordingly, the walls of the great factory town were extensively placarded, announcing the appearance of a clairvoyant at the Mechanics' Institution, on a particular evening. At the period announced, the exhibition took place, and assuredly, the case confounded alike the wise and the simple. All the local newspapers gave, in their next numbers, detailed statements; and, from the 'Manchester Guardian' we learn that, after a preliminary address by Mr. Hewes, Jack was produced in a state of supposed somnambulism, and any medical man was requested to come forward and plaster up the eyes, so as effectually to exclude the possibility of ordinary vision. A young surgeon of the place took this office upon himself, a gentleman, we are informed, whose indifference to mesmerism was only relieved by a modicum of contempt. The eyelids were bound down by adhesive plasters, folds of leather superimposed, and these again secured by other plasters; the only provision enjoined by Mr. Hewes, being that the superciliary ridge should be uncovered, as the power of vision, according to the statement of Mr. Hewes, was transferred to that region. The trials followed, and the results were truly surprising. No guessing, no hesitation, all was clearly seen indeed. In a few evenings afterwards, at another of the public institutions in Manchester, the Atheneum, a second exhibition took place with substantially similar results as on the former occasion, the plasters being fastened over the eyelids by another surgeon, associated, on the platform, with an intelligent layman for the purpose of securing complete exclusion of light. The testimony of both gentlemen was to the effect that the lad could, after what was done to him by themselves, exercise no useful vision through the eyes. Complete success once more followed. The facts were again promulgated by the 'Manchester Guardian,' and other papers, and so far all was triumphant. We have read much of the documentary testimony in favour of the reality of clairvoyance, but we do not think any of it more forcible and complete than the evidence published at this time in the newspapers; and, certainly, nothing on a superficial view of the case, could have been more so.

The matter, naturally enough, began now to excite considerable attention, and a number of literary and scientific gentlemen were anxious for a private investigation, and Mr. Hewes at once acceded to the wish. A *reunion*, accordingly, took place at the house of a medical practitioner. Without going into any detail, we can state, on the best authority, that the results on this occasion were, as usual on close examination, most unsatisfactory. About the same time, an ingenious young surgeon of the place, Mr. Dunn, made trials upon his own eyes with plasters, and then advertised that he would experimentally demonstrate and explain the way in which the success of Jack had been accomplished. At the Atheneum, the scene of previous triumphs of clairvoyance, Mr. Dunn appeared, and requested the same medical man who had officiated on the preceding occasion, to come upon the platform, and "make up" his eyes exactly as he had done those of Jack. This was accomplished; and it was stated by the medical operator that his mode of securing, by plasters, the occlusion of light, had been the same in the two instances, and, as he thought, complete and efficient. What happens? Why, Mr. Dunn, unmesmerised, was clairvoyant too!* It was stated by Mr. Hewes, who was present, that, although the thing was cleverly done, the true explanation had not been given. It has been stated that, in these experiments, the superciliary ridge was uncovered, and Mr. Dunn saw *over* the plaster from a very small crevice, having, by the exhibition of a little assumed mesmeric irritability of the muscles about the eye, contrived to bring about the formation of this aperture, after the application of the plaster strips. Whether Jack's mystic vision had been accomplished by employment of a similar manœuvre, or not, it was contended, on all hands, that no satisfaction could exist with respect to his clairvoyance, unless some other tests, more decisive than those with plasters, should disprove Mr. Dunn's position. Mr. Hewes, on whose

* Itinerant mesmerists have more than once been thus completely beaten at their own weapons, both in London and in the country. In one case, by means of a system of secret signals between two of these sham mesmerists, many of the most striking results of "the science" were produced.

good faith no suspicion whatever rested, readily agreed to any plan of closing the eyes which might be devised, compatible with avoidance of all injury to the lad. Accordingly, a few days afterwards, a party of gentlemen, medical and lay, assembled, and agreed, without the knowledge of the youth, to bind down the eyelids with shoemaker's wax, protecting the eyelashes by narrow strips of adhesive plaster. At the proper time, the assumed clairvoyant was introduced; strips of plaster were applied as usual, and, over these, a firm coating of the wax. During the application of this latter, the lad seemed to suspect what was being done, and at once exhibited a disposition to resist, but his master's influence stilled him; and the eyes were most effectually closed, and yet the pretended seat of vision, the region of the eyebrows, was abundantly exposed. But neither entreaty nor command, nor insisting, nor beseeching, were of the slightest avail in the hour of trial; Jack was inexorable, and would not even attempt to see. Mr. Hewes gave up the case. He had advertised his re-exhibition for that same evening at the Mechanics' Institution; he attended according to advertisement, announced what had taken place, and directed that all who had come should receive back their admission money; and what had been taken, on former occasions, was presented to the respective institutions where it had been received. Liverpool had, prior to this explosion, been placarded with the announcement of Jack's speedy appearance upon that stage. All was abandoned.

Now, we readily concede that, if it were certain that Alexis and Jack were arrant rogues, such a fact would not disprove the reality of clairvoyance; but when the very choicest specimens are found to be so very incomplete and unsatisfactory, does not distrust naturally arise with respect to all the rest? And ought we to admit—can we philosophically admit the truth of clairvoyance, supported only by testimony that is imperfect in itself and encompassed with suspicion?

In reviewing most of the individual cases of lucidity, even as detailed by the mesmerists themselves, there is found to be a wide discordance between them and the positive assertions, occurring in books, concerning the general fact. As an illustration of this, we extract the following account, furnished by Mr. Lynill of Manchester, from the 'Zoist' for April 1844. It is part of a communication addressed to Dr. Elliotson. It relates to the case of a young Irishwoman who, in labour, had been thrown into mesmeric somnambulism, during which no sensibility to pain was for some time apparent. A few days before labour, she had, in the sleep waking state, affirmed certain matters, suggesting the possession on her part of *intuition* and *interior prevision*. Mr. Lynill observes:

"The insensibility to pain, during an hour and a half, was complete; I believe that any operation might, during that time, have been performed without giving her the least pain. Her prediction as to the time at which she would be taken ill was verified, as she was seized soon after ten o'clock on the Wednesday night; but she was at fault in supposing her illness would not be labour. She also gave some descriptions of her child, which proved to be incorrect."

We have ascertained, as a fact, that these "descriptions of her child" included the *sex* amongst other things; and, truly, all this does not realize the expectations raised by perusal of M. Teste's account of intuition in the pregnant state, as will be apparent from the following, an extract from a previous quotation:

"One could never imagine with what tact, justice, and precision, somnambulists ascertain what passes within them .... of everything, there is formed an idea that is exact, rigorous, and mathematical .... all his estimates are of an incomprehensible exactness. .... A woman will not be pregnant eight days before she will designate the sex of her child, and *never be deceived.*"

It is not we who have rendered the last words in italics; it is the author who has done so.

Miss Martineau, whose publicly avowed adhesion to mesmerism, in its entire plenitude, has recently excited considerable attention, supplies us, in her published 'Letters,' with an instance of intuition manifested in a young girl who is designated J.; and, in the following extract, the reader has still further exemplified the kind of evidence ever brought forward in proof of lucidity:

"The next evening (Monday, October 14,) J. did not come up as usual to our *séance.* There was affliction in the household. An aunt (by marriage) of J.'s, Mrs. A., a good woman I have long known, lives in a cottage at the bottom of our garden. Mrs. A.'s son, J.'s cousin, was one of the crew of a vessel which was this evening reported to have been wrecked near Hull. This was all that was known, except that the owner was gone to Hull to see about it. J. was about to walk to Shields with a companion to inquire, but the night was so tempestuous, and it was so evident that no news could be obtained, that she was persuaded not to go. But *she was too much disturbed to think of being mesmerised.* Next morning there was no news. All day there were flying reports,—that all hands were lost —that all were saved—but nothing like what afterwards proved to be the truth. In the afternoon (no tidings having arrived) we went for a long drive, and took J. with us. She was with us, in another direction, till tea-time; and then, on our return, there were still no tidings; but Mrs. A. was gone to Shields to inquire, and if letters had come, she would bring the news in the evening. *J. went out on an errand while we were at tea,* no person in the place having then any means of knowing about the wreck; and *on her return, she came straight up to us for her séance* . . . . . . . J. was presently asleep, and her mesmerist knowing the advantage of introducing subjects on which the mind had previously been excited, and how the inspiration follows the course of the affection, asked, as soon as the sleep was deep enough, 'Can you tell us about the wreck?' J. tranquilly replied, 'Oh! yes, they're all safe; but the ship is all to pieces.' 'Were they saved in their boat?' 'No, that's all to pieces.' 'How then?' 'A queer boat took them off; not their boat.' 'Are you sure they are all safe?' 'Yes; all that were on board; but there was a boy killed. But I don't think it is my cousin.' 'At the time of the wreck?' 'No, before the storm.' 'How did it happen?' 'By a fall.' 'Down the hatchways, or how?' 'No, he fell through the rigging, from the mast.' She presently observed, '*My aunt is below, telling them all about it, and I shall hear it when I go down.*'" (p. 26.)

Without quoting *verbatim* the sequel of this story, it may be sufficient to state that the facts of the case were as set forth by J. who, on demesmerization, assumed to be ignorant of the whole affair; the news had been brought from Shields by the aunt, prior to the *séance*, it being contended, however, that J. could have no knowledge upon the subject.

Now, we do not say that here we have demonstration that J. is an impostor, and that Miss Martineau is her dupe; but the passages above, which we have placed in italics, do certainly both confirm and illustrate our position respecting the utter inconclusiveness of all such testimony. It is unnecessary to show this by any analysis; the weak points, in the account, have been exhibited in almost every notice of Miss Martineau's letters that we have seen; and we have scarcely spoken with an individual, mesmerist or otherwise, to whom the debility of this narrative has not been obvious.

We recur to the subject of clairvoyance, for the purpose of adducing one other example which strikingly shows the character and value of *lucid* phenomena, when tested in a spirit of caution and just scepticism, the absence of which, in the investigation of all such matters, must inevitably lead to the grossest credulity, folly, and extravagance. The subjoined statement is part of a communication which was addressed by Dr. Ferguson to the Editor of the Bath Herald, and which appeared in the 'Provincial Medical Journal' for Jan. 29th of this year:

*Letter from Dr. Ferguson to the editor of the Bath Herald.*

"Sir,—In the afternoon of Saturday, November 9th, Mr. Storer requested Drs. Cardew and Tunstall, Mr. Thomas Barrett and myself, to accompany him to the Daguerreotype Institution, in the Park, to see a most decisive proof of the reality of '*Clairvoyance*.' We proceeded thither, and met Mr. Freeman, the proprietor of the Institution, and Dr. Owens. A small thin book with flexible leather binding was presented to us; it was, apparently, firmly secured by thin string traversing it longitudinally and three times crossing it, in the centre and over each end, and tightly knotted. Under the cover of this book a portion of a leaf of the Park Subscription Book was placed. The "*clairvoyante*" was reported, while mesmerised (having had, at one time, a thick shawl covering her head and the book, and at another time having been permitted to retire alone with it into an adjoining light room) to have read. She subsequently, being still mesmerised, wrote down from memory what she had been able to decipher.

Copy of the "clairvoyante's" autograph description of what she read:

tion and or the donations
6 0 all down figures.
6 0 at the top park Bath.

Copy of part of the leaf read from:

Victoria Park, Bath.
Subscriptions and Donations.

| £ | s. | d. |
|---|---|---|
| 1 | 1 | 0 |
| 0 | 10 | 0 |
| 1 | 1 | 0 |
| 1 | 1 | 0 |
| 0 | 10 | 0 |
| 1 | 0 | 0 |

"While some conversation was going on Mr. Thomas Barrett went into another room, taking the book; in a short time he called Mr. Freeman, to whom he exposed the manner in which he had so removed the string at one corner, and turned back an angle of the cover of the book, that he was able to read exactly the same words, &c., as, and no more than, the child had done, replacing the string as if it had not been disturbed. Drs. Cardew and Tunstall, and myself, were shown the method pursued by Mr. Thomas Barrett; but previously the knot of the string had slipped; it, however, being situated at the back of the book, no influence was produced on the angle of the cover which was reflected. Mr. Freeman had been convinced that the '*clairvoyante*" had really accomplished something supernatural while in the mesmeric condition, that he had, before our arrival, told Dr. Owens he would consider it a point of duty to relate the fact as he then deemed it, to all the medical gentlemen he knew. My companions and myself left the Institution, but returned in a few minutes to ask Mr. Freeman if he would come forward with the book to expose the fraud practised in this instance, as we considered ourselves bound to lay the discovery before the public. He readily assented to our request, telling us at the same time that he had, during our absence, informed Dr. Owens that, being now convinced of the deception, he should certainly divulge it."

And here we cannot help making a remark on the exceeding fondness —indeed constant practice—of the mesmerists to get their clairvoyants *to read through the pages of books*, and their extreme shyness and reluctance to allow them to read through sealed envelopes or boxes. The difficulties ought to be *precisely* the same in both cases. The only explanation seems to be, that the *book* affords such palpable and obvious facilities for *cheating*, while the box and envelope prevent this. In the experiments with Alexis, witnessed by Dr. Forbes, it is certain that the young man had most ready opportunities of seeing *all the words which he read*, without any thing interposed; and in some of the cases, it is equally certain that he *did* so read them.

In selecting specimens of the evidences of *the least incredible* of the phenomena of lucidity for analytical criticism, we have conscientiously regarded those we have taken as fairly representing the remainder; and if they do so, are we not justified in maintaining that the evidence is *not* either complete or free from suspicion; and, consequently, that, as *proof*, it is grievously defective and utterly inadequate to its purpose? Still, we hesitate to say that such things are impossible. This is an epithet which is only admissible in a very limited department of science. But we persist in maintaining that, with such counter-evidence before us, we are bound, by every just principle of philosophising, to refuse credence to all testimony in support of such things which has not been subjected to a similar ordeal. And we must here remark, that it were well if this canon were kept more constantly in view, even by men of science, in the discussions on mesmerism now so general. In every company we encounter educated men of good sense and good faith, who maintain that they have witnessed these higher phenomena with their own eyes, and under such circumstances as appeared to them to render either delusion or collusion impossible. It is painful to doubt such testimony. It would be absurd to doubt it in any ordinary investigation, scientific or otherwise, as to matters of fact. But when we find, on inquiring closely of these very believers, as we always do, that in no case were means adopted to render mistake *impossible*, and when we consider that the cases above detailed, and many others equally valid, were, before detection, supported by testimony of precisely the same kind, and of equal strength, we are compelled to persevere in a scepticism which, to those who have not investigated all the bearings of the subject, and who are ignorant of its minute history, must appear at once unreasonable and unphilosophical.

In an earlier portion of this article, we proposed, as a guiding principle in these investigations, that if a new proposition were seemingly opposed to all preconceived ideas and past experience, and if it were not readily cognizable by the philosophical inquirer, it could not be admitted, unless the testimony in its favour were in all respects so decisive as not possibly to be explained away; that if any event, or series of circumstances, *may* have been in accordance with recognized facts and laws, any contrary inference with respect to the same should, provisionally at least, be deemed inconclusive. In other words, we hold it to be unphilosophical to seek for *hidden* causes, when *obvious* ones will account for an unwonted manifestation. We shall apply this rule to the further consideration of the supposed facts of lucidity, in attempting a rational explanation of some phenomena that may have given rise to these vain imaginings, as for the present we assume them to be.

Whatever may be the actual amount of folly and roguery identified with the facts of mesmerism, it is yet difficult to conceive that there have not been, occasionally, certain natural facts in somnambulism, which, by misinterpretation, have first suggested the idea of lucidity: since either a counterfeit or exaggeration presupposes commonly some actual and genuine existence as its prototype. How is it then that so many men, of undoubted honour and ability, of various countries, should, with such uniformity, through a series of years, go on with their announcements of clairvoyance, intuition, and prevision? Is it not likely that *something* has occurred to furnish at least an outline for the imagination to fill up and to colour? In the immediate sequel, we shall suggest some explanation of this difficulty; premising, however, that what we advance is mainly hypothesis, intended to furnish a possible key to the true solution; and that our remarks are offered, for the most part, as hints to those whose opportunities may enable them to test their value, by direct investigation of the real facts of the case.

We shall first inquire into the circumstances which may have originated the notion that somnambulists see at times otherwise than by means of the usual organs. Is it not that, in some instances, the sensibility of the retina may be so exalted as to enable the patient to *see* in a comparatively dark medium, one in which clear sight in the natural state would be impossible? In one of Gassendi's spontaneous cases, mentioned in a previous quotation, the somnambulist found his way readily in dark nights; but, on awaking during any of these perambulations, he was obliged to wait till daybreak from inability to see. This explanation would harmonize with the law of vital compensation; some senses in the sleep-waking state being in the most profound torpor, it is no unreasonable supposition that the *vis nervosa* may, under such circumstances, concentrate itself in the visual apparatus. This notion was entertained by Dr. Mason Good, as appears from the following observations which occur as a note in his translation of Lucretius:

"The somnambulist, or dreamer, who is accustomed to walk in his sleep, will be able to make his way towards any place to which the course of his dream directs him, with the most perfect ease, and without the smallest degree of danger. He will see as clearly, and perhaps more so, as if generally awake: yet, from the very exhaustion, and, of course, increased torpidity of the other organs, in consequence of an increased demand of sensorial power from the common stock, to support the action of the sense and muscles immediately engaged, every other sense must necessarily be thrown into a deeper sleep or torpidity than on any other occasion. Hence the ears will not be roused even by a sound that might otherwise awake him; he will be insensible, not only to a simple touch, but a severe shaking of his limbs, and may even cough violently without being recalled from his dream. Having accomplished the object of his pursuit, he may safely return, even over the most dangerous precipices, for he sees them distinctly." (Vol. ii, p. 141.)

We are aware that other explanations are suggested of the facility with which somnambulists perform many of their feats in the dark; and the ultra-mesmerist would contend that it was owing to the spontaneous development of clairvoyance. For our own parts, we take the explanation which appears to us to constitute the simplest solution of the difficulty, —exaltation of the visual sense, one which, whilst adequate to its purpose, corresponds with past experience and general analogy.

We believe that some such clairvoyance is occasionally displayed in other than somnambulistic disease. In the year 1812, Dr. James Curry, physician to Guy's Hospital, related to the Medico-Chirurgical Society the history of a case of remitting ophthalmia occurring in his own person: the account is published in the third volume of the 'Transactions;' and from this we take the following extract:

"The retina became so exquisitely sensible, that I could not bear the smallest ray of light; and I could discern every article of furniture in a room so completely darkened that other persons were obliged to grope their way in order to avoid the table and chairs." (p. 352.)

May not such exaltation of vision be developed occasionally in artificial, or mesmeric, somnambulism? And, if so, can we not understand from such a fact, how men first came to think of such a thing as clairvoyance?

We shall now proceed to speculate concerning the origin of the averments respecting *intuition*, or the attainment of information in the sleepwaking state otherwise than in the ordinary way through the senses. We are told that, when this power is developed in a high degree, the somnambulist will, amongst other things, speak and understand languages never learnt, and manifest acquaintance with sciences and individual circumstances, of which all was, and had ever been, ignorance in the waking-state. As in the case of clairvoyance, we have a difficulty in supposing that no circumstance has ever arisen, supplying, by misinterpretation, the rudimentary idea whence so wonderful a doctrine has developed itself. To those who at any time may have an opportunity of witnessing some *reputed* case of intuition, we present the following remarks and illustrations as possibly furnishing the guiding thread to the true explanation of the matter. The mesmeric notion in question may, we conceive, have taken rise from a well known psychical phenomenon which evinces itself, not only in somnambulism, but even in common dreams, and which is defined by Dr. Abercrombie as "the revival of old associations respecting things which had entirely passed out of the mind, and which seemed to have been forgotten." We will explain our meaning more at length by a few illustrations. It was our lot to pass the sweetest period of childhood with a beloved relative, whom we lost whilst in our thirteenth year. Our reminiscences of those happy hours are delightful indeed; but, whilst awake, we never can, however wishful, imagine to ourselves the exact features, and their expression, of our early friend; we do this occasionally in our dreams, yet the picture ever fades from memory on awaking, seldom remaining more than a few minutes. Again, whilst we were at a village school, when about seven years old, the noise and confusion became one day such, that the master, not knowing where to inflict the appropriate penalty, condemned every scholar to learn by heart a certain verse from the Old Testament. We have never forgotten that verse. Some years ago, however, we could not, do what we would, ascertain the particular part of scripture where this memorable verse occurred. We tried, and puzzled our head, for several days, but it was all in vain. In our sleep, one night, the problem was solved. We dreamed of Jeremiah, chapter and verse; and, on awaking, we made an immediate and eager appeal to the proper quarter, and to our surprise, at the time, ascertained that our vision had been correct. Far more striking examples than these are on record; Dr. Abercrombie, in his work on the Intellectual Powers, gives the following as having occurred to a particular friend of his, and to be relied on in its most minute particulars:

"The gentleman was at the time connected with one of the principal banks in Glasgow, and was at his place at the teller's table, where the money is paid, when a person entered demanding payment of a sum of six pounds. There were several people waiting, who were, in turn, entitled to be attended to before him, but he was extremely impatient and rather noisy; and being, besides, a remarkable stammerer, he became so annoying, that another gentleman requested my friend to pay him his money and get rid of him. He did so, accordingly, but with an expression of impatience at being obliged to attend to him before his turn, and thought no more of the transaction. At the end of the year, which was eight or nine months after, the books of the bank could not be made to balance, the deficiency being exactly six pounds. Several days and nights had been spent in endeavouring to discover the error, but without success; when, at last, my friend returned home, much fatigued, and went to bed. He dreamed of being at his place in the bank, and the whole transaction with the stammerer, as now detailed, passed before him in all its particulars. He awoke under the full impression that the dream was to lead him to the discovery of what he was so anxiously in search of; and, on examination, soon discovered that the sum paid to this person in the manner now mentioned, had been neglected to be inserted in the book of interests, and that it exactly accounted for the error in the balance."

If space would allow, or if it were needed by the argument, we could bring forward instances without number of persons in delirium, dreams, and somnambulism, accomplishing many things attempted in vain whilst in the natural state—long-lost ideas and trains of thought recovered and perfected, languages learnt in infancy but utterly forgotten, spoken once more. In some forms of insanity, certain powers of the mind will at times become unusually energetic and vivid, as exemplified in the narrative given by a patient of Dr. Willis's, after recovery. We take the following quotation from Dr. Hibbert's 'Philosophy of Apparitions,' a very curious and interesting book:

"I always," the patient relates, "expected with impatience the accession of the paroxysms, since I enjoyed, during their presence, a high degree of pleasure. They lasted ten or twelve hours. Everything appeared easy to me. No obstacles presented themselves, either in theory or practice. My memory acquired, upon a sudden, a singular degree of perfection. Long passages of Latin authors recurred to my mind. In general, I have great difficulty in finding rhythmical terminations; but then I could write in verse with as much facility as in prose." (p. 77.)

All these things to us seem reducible to one principle—morbid exaltation of some faculty or faculties, compensating, in many cases, for torpor in others; a principle in perfect accordance with analogy and the recognized laws of any physiology or mental philosophy with which we have any acquaintance.

Although acknowledging the supposition to be entitled to less consideration than the preceding, it may be questioned whether the alleged ability of mesmerised somnambulists to answer questions propounded in an unknown tongue, might, by an effort, be admitted as *possible*, on presentation of full and unexceptionable testimony to the fact, and as explicable on the same general principle. The alleged capacity *might* arise, in somnambulism, from elevation of the power by which we all, in a lower degree, become acquainted with the thoughts and feelings of another through the natural language of expression and attitude; in a word, that the comprehension, in the case supposed, exists irrespective of spoken language altogether, and comes from the tone in which they are accompanied.

We have a few words to say on the subject of *prevision*, respecting which our remarks are again offered simply as what we deem to be reasonable speculation. And, in the first place, do somnambulists ever seem to predict at all? In the course of our reading, both upon spontaneous and artificial somnambulism, we have met with some apparently well-authenticated examples, where the patients, in this condition, have announced, beforehand, the particular day and hour at which paroxysms of an epileptic or hysterical character would come on. For reasons which we shall proceed to state, we are, however, of opinion that here there has not been *prevision*, but *preordination*; that is to say, we consider the vision in such cases not to have been prophetic of the fit, but the cause of its occurrence. Many instances are on record where individuals having been told, or having dreamed, or, in some way or another, having received the impression of their speedy death, the effect upon the mind has been such as to render the foreboding effective in the production of the result. Nay, cases are narrated where persons have died at the anticipated moment, as if from the very depth and intensity of the mental anguish. A person with urgent business at some unusually early hour, will often, contrary to custom, awake exactly at that same hour. In all these results, the event is not, in our judgment, foreseen so much as it is preordained, or brought about by the anticipation itself. Precisely in this category do we place the instances of prevision, if the facts have really occurred as related. But we shall be met with the objection, that, when prevision takes place in somnambulism, the patient, in the natural state, has neither knowledge of any expected paroxysm, nor recollection of the prediction. In reply to this, we beg to observe that it is a fact sufficiently notorious, that somnambulists, though frequently oblivious of all acts done or trains of thought experienced in their sleepwaking state, do often remember all these, and refer to them as so much dreaming; somnambulism being, without doubt, some modification of an ordinary dream. Dr. Prichard furnishes us with a case in illustration, related by Horstius, as below:

> "A young nobleman in the citadel of Brenstein, was observed by his brothers, who occupied the same room, to rise in his sleep, put on his cloak, and having opened the casement, to mount, by the help of a pulley, to the roof of the building. There he was seen to tear in pieces a magpie's nest, and wrap the young birds in his cloak. He returned to his apartment, and went to bed, having placed his cloak by him, with the birds in it. In the morning he awoke, and related the adventure as having occurred in a dream; and was greatly surprised when he was led to the roof of the tower and shown the remains of the nest, as well as the magpies concealed in his cloak."

Similar statements and cases are given in accounts supplied by writers on superinduced, or magnetic somnambulism; and, as will be seen from a previous extract, Dr. Passavant relates a case where "it depended upon her will to *remember or not*, on awaking, these interior observations." Still it may be said that the parties themselves evince total ignorance of any prediction of an expected fit. We must here remind the reader that when we have to deal with anomalously-hysterical persons, there is always reason to suspect deception; and what is more likely to lead to the manifestation of this characteristic than an expected attainment of a reputation for something verging on prophecy? Yet may it be contended that fear or anticipation will not lead to the result; this last objection will hardly arise in a medical quarter. Dr. Copland states: "I have no doubt of the fit being often renewed at pleasure, almost as readily as tears may be shed by recalling or adverting to various feelings, emotions, or circumstances;" and adds, "I have seen instances which have convinced me of the fact." Under all these circumstances, then, and in the above view of the case, we reiterate our hypothesis, that if anything like *prevision* ever do occur in somnambulism, it is *preordination.*

In the impossibility of speaking from the facts of the case, we shall not enter into the possible mode of explaining away other magnetic wonders,—the higher order of lucid phenomena—such, for instance, as intuitive knowledge of remote and long past occurrences, of the bodily and spiritual condition of persons at a distance, and unknown when awake, the state of things in the stars, and the like, because little, we think, need be said with respect to them, except that *they are incredible.* We may, or may not, admit their possibility as *natural* facts; that is a separate question: independently, however, of any conclusion to be drawn from the wild and extravagant character of the statements themselves, the evidence, on which they rest is grievously defective in every just requirement; which circumstance, if space would allow, might readily be demonstrated.

Here we may not inappropriately observe that a point where the ultra-mesmerists have ever seemed to us most weak and assailable is this: they bring forward what, at first sight, seems good evidence of certain wonders which, with some difficulty, one may allow to be *possible;* but then the same evidence is adduced in support of what few besides themselves could at all admit to be so, excepting as a miracle. As an illustration of our meaning, the following example occurs to us. We take the account from Dr. Elliotson's paper, on Alexis, in the January number of the 'Zoist,' for the present year. A gentleman had been bitten by a dog; the fact was never revealed to living mortal; the wound had long since healed; a scar only remained. The gentleman visits Alexis who, on being questioned, *sees* the scar although covered by the clothes, and declares the fact of there having been a wound. Let us concede the credibility—at least the possibility—of the story thus far. But the history goes on to relate how, when Alexis was asked respecting the *origin* of the wound, its entire history, although of years long past, becomes revealed!

"If not by ball, sword, or bayonet, how was it done?" A long, very long pause. At length a sudden light appears to stream in upon him. He begins in a low tone, as if muttering to himself. 'Oh, I see; yes, you get off your horse; you open the yard gate; the house is so and so; you cross the yard; you go to ring at the bell—(he becomes quite excited)—Oh! there it is. *It* comes jumping and barking towards you; *it* is such a colour; *it* jumps on you; *it* seizes you; *it* bites you here," pointing to the hip! imitating the dog and all its movements; "it is, it is, (he is so agitated with the vivid scene that he cannot get at the word, or he sees obscurely, at last he gets out) it is a great dog!" (p. 495.)

Thus it is, in the whole of this matter of lucidity; the witnesses are as good, positive, and numerous, in attestation of the very wildest stories, as of those which draw less largely upon our credulity. Does not this circumstance destroy the value that might otherwise attach to some of the evidence?

Still, that persons of undoubted ability and scientific attainments should continue to attest the reality of lucid somnambulism, is to many persons a subject of serious perplexity; and the question recurs: can it, after all, be otherwise than true? Do not these men—honest and clever—profess to have seen it? Here *is* a difficulty, without doubt, but only a minor one. As Cabanis wisely observes, there are certain errors of which only men of talent are susceptible. The reason of which fact may probably be found in the ardour of imagination which characterizes some individuals of high mental endowment. Enthusiasm, inquisitiveness, and eagerness for novelty, hurrying such persons to premature conclusions, they seem to cling to these, as sources of wonder and delight, with most affectionate tenacity. But, indeed, in all ages, a love of the marvellous has originated follies without number; follies which are the peculiarity of no one class. They are often conceived by able, curious, and imaginative minds, and the multitude receive them on trust. The ancients had their oracles and their auguries, in which some of their very sages had confidence; later times have witnessed a belief in witchcraft not only by the ignorant and uneducated, but by many among the learned and accomplished, such men, for example, as Sir Matthew Hale. A belief in the *second sight* of Scotland was once most general through large tracts of country, and its reality was credited by persons whose judgment was and is respected upon most other subjects. Amongst these was no less a personage than Dr. Samuel Johnson. Multitudes of individuals, for ages, testified to the miraculous efficacy of the Royal touch. We assert fearlessly that the power of clairvoyance is not at this time so well supported by evidence as that of the Royal touch once was—or even that of riding through the air on a broomstick. Yet, all these things are now discredited; and why are they so? Plainly because their seemingly preternatural character demands for the proof an *extraordinary* kind of evidence which the recorded accounts do not exhibit; although they are by no means deficient in *ordinary* evidence. Hale for Witchcraft, and Wiseman for the Royal touch, were authorities, in their day, certainly equal to Elliotson and Townshend at the present time. Why should we yield assent to the latter, and not to the former? We know that it has been the fashion to claim for the nineteenth century a comparative freedom from the folly of credulity; but we very much think that our superiority in this respect equals not the vaunt; we suspect that, however the *objects* may change, the *disposition* remains but little diminished.

> "Der kleine Gott der Welt bleibt stets von gleichem Schlag,
> Und ist so wunderlich als wie am ersten Tag."—FAUST.

But, in point of fact, what says the history of days not remote, nay of the very day in which we live, to this vaunt of superiority? The following is a true chronicle of witchcraft and its fortunes, in the last few years of the seventeenth century, as minutely detailed, with all the names and circumstances, in Dr. Hutchinson's 'History of Witchcraft.'

1691. Three women committed for witchcraft; one died in gaol, two tried and acquitted. "Several tried by swimming, and some drowned in the trial."

1692. "Nineteen hanged at Salem in New England; one pressed to death; eight more condemned; fifty confessed themselves witches and were pardoned; one hundred and fifty were in prison."

1693. Two women committed in Suffolk; one died in prison, the other tried and acquitted.

1694. A woman tried at Ipswich and acquitted.

1695. A woman tried at Launceston and acquitted.

1696. A woman tried at Exeter for bewitching children. "The mother of the children deposed that one of them walked up a smooth plastered wall, till her feet were nine foot high, her head standing off from it." Notwithstanding this evidence and the equally attested fact of her having "a nipple on her shoulder which was sucked by a toad," the woman was acquitted.

1697. "About eight and twenty were accused by a girl eleven years old, in the county of Renfrew in Scotland. One old man died in prison; another was found hanged in gaol; five saved themselves by confessing, and upon their testimony seven were executed."

1698. A woman was "tried at the Old Bailey, and set in the pillory, for pretending to be possessed. About eleven in all have been tried and acquitted."

1700. A woman tried at Guildford and acquitted.

Nay, little more than a hundred years ago, in 1716, in the palmy days of English literature, in the days of Addison and Pope, a woman and her daughter (aged *nine*,) were hanged at Huntingdon for selling their souls to the devil! (See reprint of 'Tryal of Witches,' in 1664, p. 28.)

Barrington, in his observations on the Statute 20, Henry VI., estimates the number of persons put to death in England for witchcraft at 30,000.

It is only about twenty years since, that the highways of Sussex and Hampshire, and the passage-boats from Southampton and Portsmouth were daily crowded and loaded by persons thronging to the Isle of Wight to be cured by the *touch* of a common sailor!

In a work published by a British peer in 1841,* we have marvels recorded as then being transacted, and authenticated by numerous witnesses, which emulate the loftiest legends of the middle ages, and some of them in direct opposition to the laws of physics. And at this very moment there is a German bishop instituting solemn festivals in honour of the discovery of the sacred vestment (there shown) actually worn by Jesus Christ!

Nay, more marvellous still, in the year 1840 we find a British physician and one or two surgeons vouching for the truth of direct communications from Heaven, both by word and deed, vouchsafed openly, by day and night, to a poor ignorant girl of Sunderland.

We have not room to transcribe the noises, songs, prayers, scriptural exhortations, &c. described by this narrative to have been heard by all concerned; but we will extract a few instances from Dr. Clanny's own narrative of "facts," just to give the reader a taste of things credited even by doctors of physic in this our land and this our day.

"At our next interview we became more intimate, and I asked her why she was so backward with me at our first meeting after her recovery? Her reply was very remarkable; she half whispered to me in a childish voice, 'You were a

* Letter from the Earl of Shrewsbury descriptive of the *Estatica* and *Addolorata*; London, 1841.

stranger to me, for I had never seen you before that time, and I saw an angel standing at your back.'

"I took an opportunity of asking whether she could give me a few texts from the holy Scriptures, to which she immediately assented. Upon taking a slip of writing paper, and a pencil from my pocket, she stood firmly before me, and looking upwards, gave the following texts, as rapidly as I could write them down.

"Psalm lv, 17; Psalm xcix, 2; St. Matthew, xxviii, 15; 1st Corinthians, the whole of the xv chapter; 2d Corinthians, the whole of the xiii chapter; 2d Thessalonians, ii, 14, 17, inclusive; Hebrews, ii, 15; 2d St. John, 4, 5; Revelation, xxii, 12, 18, inclusive.

"Some days afterwards I inquired of Mary Jobson, how it occurred that she had the texts so ready; when she informed me, that about half an hour before my arrival at her father's house, a voice informed her of my intended visit, and that I would ask her for some Scripture texts. I then took leave to ask her to inform me whose voice it was, when she instantly replied, that the voice commenced to speak in the following words, 'I am the way, and the truth, and the life: no man cometh unto the Father but by me.' This I knew to be the language of the Saviour of the world, as we find recorded in the New Testament. I then asked her whether my name had been mentioned by any other voice; when she informed me that such was the case, at two different periods, at one time by Saint Paul, and at another period by Saint Peter.

"I asked Mary Jobson to oblige me by informing me whether, as I conjectured from her fixed attitude, she read the texts which she gave me on that momentous occasion, in the atmosphere. Her reply was, that a figure clothed in white apparel, having a somewhat dark complexion, stood before her, and pronounced the texts to her in a deliberate manner whilst I wrote them down: I need scarcely add that I was not permitted to see the figure, or to hear the words spoken.

"I may here mention, that soon after Mary Jobson was restored to health, her mother showed me the figures of the sun and moon, upon the ceiling of the room in which her child lay for so many weeks; and though her husband, in his state of unbelief, had white-washed over these figures, nevertheless they were still very distinct, and appeared to me to be most accurate in their outlines.

"If any man presume to say that unembodied spirits can have nothing to do with us, and that they are not identified with human affairs, I will fearlessly tell him that he speaks like an idiot, and begs the question like a false reasoner."*

In reading these and other accounts of preternatural visitations to man, and of the possession by man of superhuman powers, it is impossible not to be struck with one remarkable feature which obtains in all, viz., the lamentable poverty of the performance, after such a magnificent overture. The paltry knot that is undone is ever most unworthy of the fingers of the god; the ridiculous animal produced, is invariably a disgrace to the labouring mountain.—The devil appears—and lo! a poor, half-starved, old woman sells her soul to him, merely to get a pennyworth of her neighbour's milk, or to have a ride on an uncomfortable broomstick. The saints and angels, and even the higher powers, come down from heaven to Dr. Clanny's patient—to do what?—to give a few run-away knocks at a door,—to twirl a bit of pasteboard on a chimney-piece,—to scratch pictures of the sun and moon on a dirty ceiling,—or, at most, to sing unintelligible hymns with a Northumbrian burr. In like manner, our clairvoyants, instead of settling for ever, as they might easily do, all our doubts and difficulties in astronomy, geology, chemistry and physiology;—instead of unfolding the secrets of the cabinets of kings and prime ministers, and thus benefiting their country, or making their

* A Faithful Record of the Miraculous Case of Mary Jobson. By W. Reid Clanny, M.D. F.R.S.E. M.R.I.A., &c. Second Edition. Newcastle-upon-Tyne, 1841.

own or their masters' fortune by forestalling Rothschild or Moses and Son in the purchase of bank-stock or railroad shares; nay, instead of even doing such little matters as the hazel-rod diviners used to do, the pointing out the presencc of a nice spring of water or of a productive tin or copper mine, these provoking people will persist in putting us off with such feats as counting the pictures in our dining-rooms, describing the colour of night-caps and doublets, laboriously misspelling words inside rumpled letters, or on gold rings in jewellers' cases,—and a thousand other things of equal importance, and which we all knew before. We have never yet heard a satisfactory explanation of this perversity of the seers. It is in itself almost enough to make their best friends cut their acquaintance. At any rate, we are resolved that when WE have the good luck to have a clairvoyant of our own, we shall not be so humble-minded as some people.

After such details as we have just given, have we any right to boast so much of our superiority in matters of belief over our predecessors? Or is it very unlikely that the men of the next century may look back to some of *our* doings with the same wonder that we regard the recorded marvels of the last century? Nay, to say nothing of the ready credence afforded by so many to the greatest extravagances of mesmerism,—the actual state of religious belief and practice among large bodies of persons in this country, would give countenance to the expectation that the doctrine of *possession* at least, if not witchcraft, might ere long be revived once more, and flourish in all its pristine glory. There seems no good reason for thinking that the remark of her Majesty's chaplain in the last century might not be equally applicable in the present. "I make no great doubt," he says, "but that we have as many devils now amongst us as they had in other ages; for we have as many temptations, and lies, and thefts, and adulteries, and murders, that are the devil's works; but our witches, for the present, are gone after the poet's gods and modern fairies. But I must add, that they are not so far from us, but that, if we should have a prince, and judges and juries, and witch-finders, of the same principles, that found out so many before in two years' time, in all probability they would find as many now."*

In the whole subject of somnambulism, there is, undoubtedly, truth sufficient to engage the regards of the curious; enough that is strange and difficult of explanation to dazzle, and to excite the wonder of the enthusiastically imaginative; and occasionally the most gifted are brought, by a gradual process, to sacrifice at the shrine of Lord Bacon's *idola theatri,* which, in the language of the great father of modern philosophy, have gained influence over the mind "from the perverted laws of demonstration." There is a fund of wisdom applicable to our present argument in the following passage, which we take from the 'Novum Organum:'

"When the mind is once pleased with certain things, it draws all others to consent, and go along with them; and though the power and number of instances, that make for the contrary, are greater, yet it either attends not to them, or despises them, or else removes them by a distinction, with a strong and pernicious

* An Historical Essay concerning Witchcraft. By F. Hutchinson, D.D. Chaplain in Ordinary to Her Majesty. London, 1718, p. 50.

prejudice to maintain the authority of the first choice unviolated. And hence, in most cases of superstition, as of astrology, dreams, omens, judgments, &c. those who find pleasure in such kind of vanities, always observe where the event answers, but slight, and pass by the instances where it fails, which are much the more numerous."

The following passage from Mr. Townshend's 'Facts of Mesmerism,' suggests the direct application of this philosophy to the matter now under discussion; it looks very like *confessions*, as a perusal of the Reverend author's entire work forces the inference that he is himself the great sublime he draws:

"There is in the sensations of him who finds that he is capable of exercising the mesmeric influence that peculiar charm which ever waits upon the development of a new faculty. Even the swimmer, who learns at length to surmount the boisterous surf, or to stem the adverse stream, will revel in the consciousness of awakened power. How much more must the mental enthusiast riot in the display of energies so long concealed, so wondrously developed! Self-love adds her flattering lure to the attractions of novelty; the pride of exerting an influence over others awakens in his breast. It is he himself who is the author of his own enjoyment; and the fairy scenes appear to him fairer still, because they are of his own creating. Unexpectedness, too, that principal ingredient of pleasure, yet more entrances and bewilders the astonished novice, who perceives such mighty effects resulting from his employment of a few and simple means. He feels that he is 'greater than he knows,' and he advances into the yet unconquered province that lies before him, with all those alternations of rapture and surprise which agitate, yet please the explorer of strange regions. He trembles—he hesitates—he catches a glimpse of a new prospect. ........ Thus excited, how shall we subside into the calmness, wherein alone the mind can compare, can select, or reject? How, from the very heights of contemplation, shall we stoop to the observation of petty facts—to the detection of fallacies—to the routine of ordinary examination? Deeming that we know all things by intuition, how can we condescend to reason? Rather are we tempted to frame some vast and all embracing theory, to rush at every subject, and, consequently to fail in all. ... The barrier of prudent hesitation is cast down, and his mind is left a prey to the invasion of every idle fancy. That he was once even of a sceptical spirit will profit it nothing. Rather, with that propensity to rush into extremes which is a part of human imperfection, the more incredulous he has hitherto been, the greater will be his rebound to credulity." (p.24.)

We here close our review of the general character of the facts of mesmerism. A few words will suffice to recapitulate the affirmative inferences to which we have already come respecting them. They are these: The phenomena in question are, in some instances, just what they seem to be, states of disease, hysteria or somnambulism, superinduced by artificial agency, yet constituting under such circumstances no pathological *speciality*. Whether such abnormal conditions of the system arise from inappreciable or internal causes, and then called *spontaneous;* or whether they are brought about by obvious, outward, and designed agency, and, in that case styled *mesmeric;* they are essentially the same hysteria, and the same somnambulism, differing only in their etiology.

It now only remains for us to discuss the theories which have been proposed in explanation of the facts here admitted. What is mesmerism? By what agency are such curious states of the system accomplished? In the language of the Rev. Mr. Townshend, what is the mesmeric medium? Do the effects, according to the more prevalent theory, come from the transmission of some vital fluid to the patient from the person of the operator? Or, is it that an impression is made upon the patient, through some all-pervading ether, by the will or by the gestures of the magnetiser, according to the original notion of Mesmer himself? In one word, is mesmerism, in the language of Dr. Elliotson, owing to the activity or operation of some *occult* influence possessed by one animal body over another?

These considerations suggest materials for an inquiry of great interest and importance; and into this, as a pure matter of evidence, we now propose to enter; guided by the same principles as those by which we have been directed in the other divisions of this investigation.

If the mesmeric effects can in all instances be shown to follow upon *sensible* impressions, any theory, we apprehend, which involves an *occult*, hitherto unrecognized influence, need not be admitted. In the interpretation of all complicated facts, simplicity must prevail, and old principles be drawn upon when adequate to the solution, and new ones be assumed only in the opposite case. Now, every well-informed mesmerist will readily grant that all the results accomplished by the presumed medium, or occult agency, may arise independently thereof; further, that by influencing appropriately, and through the senses, the moral and physical state of the subject, mesmeric phenomena will develope themselves; and that whether, or not, imagination (this term being employed in its widest sense) always brings about the characteristic conditions, it is potent enough to do so in many cases. We shall adduce a few facts in demonstration of these propositions.

M. Bertrand, in the course of his work on animal magnetism, gives the subjoined recital, showing the power of imagination in the induction of somnambulism:

"I had, amongst others, a female somnambulist who exhibited very curious phenomena, of a character not to be doubted, (one of these was an absolute insensibility to every kind of excitement.) Being after some time compelled to be absent, I left her in the hands of one of my friends, who was very anxious to continue the treatment. Up to that time, I had heard a great deal said of the substances to which the magnetic virtue was communicated . . . . not having faith in all that was told me, I had paid no serious attention to this matter; later on, the perusal of a great number of works, and my conversations with magnetisers who doubted of nothing, suggested to me to make trial of this power, and to see if I could not influence my somnambulist, in spite of the distance of a hundred leagues which separated me from her. I wrote in consequence to my friend, and sent to him a little magnetised note, which I prayed him to place upon the stomach of the patient; I indicated the epigastrium, because I had always heard this locality mentioned in these experiments. The experiment was made; it succeeded, and the patient had a sleep accompanied by all the customary phenomena. However, I did not conceal from myself that the patient having been apprized of the experiment which we were anxious to try, it might be that the sleep, although quite real, had been produced by her imagination alone. I made then another trial to know what to make of it. I wrote a second letter which I did not magnetise, and I sent it as if it had been magnetised, warning the patient that it would cause her to fall into somnambulism. In fact, she fell into this state, which even this time presented all the characters which had been usual."

"I communicated this result of my experiment to the magnetisers whom I frequented; they appeared greatly surprised thereat, and, not being able to recognize the power of imagination in a manner so marked, they pretended that if the last letter had produced the effect which I said, it was only because, in writing it, I had (even without the intention) impregnated it with my fluid. I set about an experiment which should teach me what to think of the thing. I asked one of my friends to write a few lines in my place, and to strive to imitate my writing, so that those who should read the letter should mistake it for mine, (I knew he could do so.) He did it: our stratagem succeeded, and the sleep was produced just as it would have been by one of my own letters."

Mr. Braid, of Manchester, who is understood to have devoted much attention to this subject, states, in the course of his work on "Neurypnology," that he produces all the genuine phenomena of mesmerism, more rapidly, more surely, and more uniformly, than the animal magnetisers do, by his process of "hypnotism,"—a designation applied by himself to his peculiar procedure, one in which the patient is set to mesmerise himself. We extract the following from Mr. Braid's book:

"Take any bright object (I generally use my lancet case) between the thumb and fore and middle fingers of the left hand; hold it from about eight to fifteen inches from the eyes, at such position above the forehead as may be necessary to produce the greatest possible strain upon the eyes and eyelids, and enable the patient to maintain a steady fixed stare at the object. ...... It will generally be found, that the eyelids close with a *vibratory* motion, or become spasmodically closed. After ten or fifteen seconds have elapsed, by gently elevating the arms and legs, it will be found that the patient has a disposition to retain them in the situation in which they have been placed, if *he is intensely affected.* If this is not the case, in a soft tone of voice desire him to retain the limbs in the extended position, and thus the pulse will speedily become greatly accelerated, and the limbs, in process of time, will become quite rigid and involuntarily fixed. It will also be found, that all the organs of special sense, excepting sight, including heat and cold, and muscular motion, or resistance, are at *first* prodigiously *exalted*, such as happens with regard to the primary effects of opium, wine, and spirits. After a certain point, however, this exaltation of function is followed by a state of depression far greater than the torpor of *natural* sleep. From the state of the most profound torpor of the organs of special sense, and tonic rigidity of the muscles, they may, at this stage, *instantly* be restored to the *opposite* condition of extreme mobility and exalted sensibility, by directing a current of air against the organ or organs we wish to excite to action, or the muscles we wish to render limber, and which had been in the cataleptiform state. By mere repose the senses will speedily merge into the original condition again." (p. 27.)

Mr. Braid gives examples of somnambulism, and other unwonted conditions of the nervous system, resulting from the employment of *hypnotism*, just as we read of these things in the mesmeric books; and he appears, in some of his experiments, to have taken away the possibility of any magnetic emanation from himself, by leaving the apartment in which was the subject of experiment, after having given the appropriate directions regarding the fixed stare. Mr. Braid has exhibited his method, not only in London and Manchester, but in several of the principal towns in the kingdom. On one occasion, we, ourselves, saw nearly twenty persons all staring at a cork bound on the forehead of each; and, in almost every case, was there an effect, real or simulated, analogous to that which obtains in more purely magnetic operations.

Mr. Catlow, also of Manchester, a sort of rival, it seems, of Mr. Braid, in this mesmeric affair, has lectured extensively on this subject; and, according to the published accounts which we have seen, this gentleman produces sleep and the other phenomena, by slowly and soothingly brushing the forehead with a soft brush, attributing the effects to continuity of gentle impression upon one or more of the senses, coincidently with concentration of attention, on the part of the patient, to the process going on. Mr. Catlow, we know, has induced by his method genuine mesmeric states, (allowed to be such by zealous magnetisers,) although he himself repudiates altogether the idea of exoteric animal influence.

In further exemplification of mesmeric effects, without animal magnetism, we shall record some of our own small doings in this line. We were one evening in company with a young lady who, we had been informed, had, under operation, evinced high mesmeric susceptibility. We requested permission to test this ourselves, and were obligingly permitted to do so. Accordingly, we commenced to *magnetise* the lady, by maintaining the thumbs in apposition with those of our subject, and fixing the gaze at the same time upon her eyes, with all the intensity our will could command; in a few minutes, a sort of hysteric somnolence ensued. Having satisfied ourselves thus far, we demagnetized. We next proceeded to *hypnotize* the same lady, adopting Mr. Braid's mode of directing the stare at a fixed point: the result varied in no respect from that which had taken place in the foregoing experiment; the duration of the process was the same, and its intensity of effect neither greater nor less. Dehypnotization again placed us where we were. And now we requested our patient to rest quietly at the fireplace, to think of just what she liked, and look where she pleased, excepting at ourselves who retreated behind her chair, saying that a new mode was about to be tried, and that her turning round would disturb the process. We very composedly took up a volume which lay upon a table, amused ourselves with it about five minutes, when, on raising our eyes, we could see, by the excited features of other members of a little party that were assembled, that the young lady was once more *magnetised.* We were informed by those who had attentively watched her during the progress of our little stratagem, that all had been, in every respect, just as before. The lady herself, before she was undeceived, expressed a distinct consciousness of having felt our unseen passes streaming down the neck.

It may be contended that, although mental impression and isolation of sense may often induce mesmeric states, the existence of another agency capable of doing the same thing is not disproved ; just as the ability to create a diarrhea in some cases by sudden fear, in most by jalap, does not discredit the efficacy of croton oil. Very true ; but, in ordinary magnetic experiments, the assumed agency, the occult influence, stands not alone; it is in conjunction with other conditions of proved efficacy in the production of the effects in question. If the foregoing facts, selected from multitudes of a like kind, be admitted, it must be conceded that no experiment can prove the exercise of any foreign vital influence, but a successful operation upon an individual who has no knowledge of what is going on, nor even a thought at the time bearing upon the matter: the result must come wholly unanticipated.

Examples, however, of magnetic operations with successful results upon patients near and at a distance, are recorded, where they themselves have been altogether unacquainted with what was intended, where, by the unexpressed will of the operator, sleep and other effects have been induced on the part of subjects intimately *en rapport* with their magnetisers. After the copious details on other branches of this inquiry which we have already given, we have no space for the analysis of these instances with a view to detect the possible source of fallacy; nor, indeed, do we think it necessary. Considering the extraordinary credulity and excitable enthusiasm of the professed animal magnetisers; knowing, moreover, the little care and precaution which are commonly taken to shut out any probable error or mistake; we candidly acknowledge that we receive all these accounts with the greatest suspicion. Of course, when men like the Rev. Mr. Townshend attest these things, (and this gentleman, in his book, gives several instances of unconscious mesmerism,) we have every confidence in the *integrity* of the narrator; but, on grounds which our limits will not allow us to submit, we think that due measures had not been taken to secure an absence of all anticipation of a coming effect. Besides a distrust in the general accuracy of mesmeric details, we subjoin certain reasons of our own, resting upon experience, for discrediting these particular stories.

On one occasion, M. Lafontaine advertised that he had a female subject in whom he had induced, and could again induce, sleep, whilst he was in another room, and without her consciousness. We attended this exhibition. Certain precautions were taken, and the experiment was confessed, by M. Lafontaine, to have failed. About two years ago, an intelligent and a well-educated friend of ours had a female servant whom he had repeatedly thrown into a sleepwaking state, and on whom he had tried a variety of experiments, many of which we ourselves witnessed. We were at length informed that he had succeeded in magnetising her from another room, and without her knowledge; that he had paralysed particular limbs by a fixed gaze unseen by the patient; and we hardly know what besides, These things were circumstantially related to us, again and again, amongst others, by the medical attendant of the family, a most respectable and intelligent friend of our own. We were yet unsatisfied: we considered that these experiments were so constantly going on, parties were so often going up to the house to witness these things, that the presence of a visitor, or the occurrence of anything unusual, was sure to excite expectation of some mesmeric process. We were invited to come and judge for ourselves, and to propose whatever test we pleased. Now, had we visited the house, we should have felt dissatisfied with any result, and we therefore proposed the experiment should be made at our own residence; and it was made under the following circumstances. The gentleman early one evening wrote a note, as if on business, directing it to ourselves; he thereupon summoned the female servant (the mesmeric subject) requesting her to convey the note to its destination, and to wait for an answer. The gentleman himself, in her hearing, ordered a cab, stating that if any one called he was going to a place named, but expected to return by a certain hour. Whilst the female servant was dressing for her errand, the master placed himself in the vehicle, and rapidly arrived at our own dwelling. In about ten minutes afterwards, the note arrived, the gentleman, in the mean time, being secreted in another apartment. We requested the young woman, who had been shown into our study, to take a seat whilst we wrote the answer, at the same time placing the chair with its back to the door, which was ajar. It had been agreed that, after the admission of the girl into the place where we were, the magnetiser, approaching the door in silence, should commence operations. There then was the patient, or subject, placed within two feet of her magnetiser, a door only intervening, and that but partially closed, but she, all the while, perfectly free from all idea of what was going on. We were careful to avoid any unnecessary conversation with the girl, or even to look towards her, lest we should raise some suspicion in her own mind. We wrote our letter as if in answer for nearly a quarter of an hour, once or twice only making an indifferent remark, and, on leaving the room for a light to seal the supposed letter, we beckoned the operator away, and, returning, sealed the note, and dismissed the young woman, as magnetically charged as when she arrived. No effect whatever was produced, although we had been told that two or three minutes were sufficient, even when mesmerising from the drawing-room, through walls and apartments, into the kitchen. In our own experiment the intervening distance had been much less, and only one solid substance interposed, and that not completely; but here, we suspect, was the difference—the subject was unconscious of the magnetism, and expecting nothing! Another friend of ours informed us that he had, on two or three occasions, induced powerful mesmeric effects, operating in a room separate from that in which the patient (a young woman also) remained, and quite unknown to her. Ever ready to be convinced of the reality of these assumed facts, we agreed, upon our friend's invitation, to investigate the matter, proposing, however, a test of our own, and one calculated, as we conceived, to meet the exigencies of the case. Total failure once more followed.

These negative facts cannot, of course, settle the point; one positive and sure instance would more than counterbalance any amount of experience such as ours. We have adduced the above circumstances, because, whilst they exhibit the grounds of our own scepticism, they may furnish hints to others cautiously testing professed cases of unconscious mesmerism. And here, as in lucidity, we must insist that the evidence in support of their reality, involving, as they do, something that is opposed to the common consent of mankind, must be complete, and beyond suspicion, and the result absolutely unsusceptible of any other construction.

We shall but briefly notice some other circumstances which are often referred to as proof of some mysterious communication between the operator and subject, and which are thus considered to prove the *objectivity* of mesmerism. One of these is the absence of all regard or attention on the part of the magnetisee, to any one but the magnetiser, or some one with whom a *rapport* has been established. This, however, is only an occasional and apparently incidental phenomenon. We ourselves see nothing in this matter but a manifestation of the well-known circumstance of the sleepwaker being "awake to objects of attention, and torpid to things at the time indifferent." In his bed, the wearied accoucheur can sleep, in spite of thunder and other noises, but the slightest tinkle of the night-bell he at once recognizes; in the *rapport magnétique* we do but see an analogous fact, illustrated, also, in one of the examples afforded of spontaneous somnambulism, that of Castelli, in another part of this article. It has been suggested that some exoteric vital influence must be at work in *demagnetization*, seeing that the patient, having evinced insensibility to severe excitation, cannot be supposed to feel the slighter shock of currents of air, and other means of an equally simple kind, employed by the mesmerists to recover their subjects. These things, however, will receive a partial explanation in the anomalous and *peculiar* sensibility of many hysterical persons, sometimes displayed in the spontaneous affection. A case was recently related to us of a lady in hysteric disease, who was quite unmindful of many strong impressions, but became powerfully excited by the contiguity of a fly in motion. This was attributed by our informant to the current of air produced by the insect, as the patient evinced in other respects a high sensibility to the slightest atmospheric current. It happens often in spontaneous somnambulism that patients will resist numerous energetic measures resorted to for the purpose of awaking them, whilst some seemingly indifferent action will bring the thing about. We presume this to be dependent upon some curious relation between the outward impression and the inward anomalous *dream* of the sleepwaker. Thus, Muratori relates the case of one Agostino Torari, who could only be awaked in two ways, by tickling the feet, or by sounding a horn in the ear; and, by reverting to Smellie's case, it will be noticed that, however uninfluenced by many kinds of severe excitation, the patient was at once aroused by compression of the wrist coincidently with a call by name.

Traction of the magnetisee by the magnetiser has been much adduced as *proving*, if true, the theory of animal magnetism. Mr. Catlow's experiments, however, suggest that, in some cases at least, this traction is owing to the subject being sensible to the approach of warmth, even though unacted upon by other and stronger excitants. Heated irons are held in the direction of, and at some distance from, some part of the patient's body, and a movement takes place towards the source of what may constitute an agreeable feeling. The hand being cold, in Mr. Catlow's cases, exerts no attractive influence; heated, however, the effect comes. We have seen these experiments repeated, and to us they have seemed genuine. From certain experiments that we have seen we have reason to adopt several other explanations of this phenomenon, varying with particular cases, but our limits will not allow of any prolixity upon this theme. We must here, however, contend that an experiment to *prove* an occult mode of attraction must exclude sensible impressions. Dr. Darwin, in the first volume of the 'Zoonomia,' relates the following case, a *spontaneous* one, of peculiar sensibility to heat, with general insensibility. It is set forth in a letter from Dr. R. W. Darwin, having been written when this latter was a student in Edinburgh:

"A man who had lately recovered from a fever, and was still weak, was seized with cramps in his legs and feet; which were removed by opiates, except that one of his feet remained insensible Mr. Ewart pricked him with a pin in five or six places, and the patient declared he did not feel it in the least, nor was he sensible of a very smart pinch. I then held a red hot poker at some distance, and brought it gradually nearer, till it came within three inches, when he asserted that he felt it quite distinctly."

We suspect that when metals, magnetised, induce particular effects (if they ever do), it is occasionally owing to the recognition of the heat imparted in the process, and, at other times, to the imagination.

We have heard it contended, nevertheless, that community of sensation between the magnetiser and magnetised constitutes proof demonstrative of some occult link between the parties, and thereby establishes the existence of animal magnetism as a distinct agent or principle. From the accounts published of this supposed phenomenon, it seldom seems to go beyond the sense of taste; and a simpler explanation of the fact itself is suggested to our mind. In a case of this kind, which we had opportunities of carefully observing, the female subject, we had every reason to believe, was truly in the sleepwaking state; amongst other evidences of this condition, we may adduce the immobility of the widely-dilated pupil on the approach of light. The mesmeriser caused confectionary, wine, milk, water, and fruit to be placed upon a drawing-room table (this investigation took place in a private family); the various articles were tasted, and the girl generally imitated the action, and, in the majority of cases, her expressed sensations of taste corresponded with those of her magnetiser, but not always. On a second occasion, we placed on the tongue of the latter, unknown to himself, a small portion of the sulphate of quinine; no recognition whatever of this was made by the magnetisee: we had come prepared with quinine, a substance, we conceived, the taste of which would neither be imagined nor anticipated. A third time, a gentleman, whose functions had been judicial, joined us in the observation of these experiments, and took upon himself their guidance. All being ready, our learned friend directed the magnetiser to drink the water; this, we believe, was, after some questioning and delay, recognized by the patient. Milk was next taken up, but our barrister friend interdicted it by a sign, and once more pointed to the water; not water, however, but something else was alleged to have been tasted by the somnambulist. Our friend perseveringly indicated the water as the object of potation; various articles on the table, however, were stated by the girl to have been tasted in succession: and the affair was confessedly a thorough failure. What probable inference was to be deduced from the foregoing? Our own was, that the sleepwaker, characteristically attentive to the operator, felt impelled to imitate his actions, of which, as we conceive, she had some sort of perception, obtained, possibly by exaltation of the sense of hearing, and when questioned as to what she tasted, there might be a species of *dream* that she tasted what she supposed the magnetiser to have tasted. She never could have conceived that quinine was placed upon his tongue, and she did not recognize its taste; nor, after what had taken place on former occasions, would she ever dream that water, time after time, would be the material for experiment.

Mr. Newnham, whose work has just reached us, has a passage which corroborates our notion, that community of taste is a mere *fancy* on the part of the sleepwaker.

"A similar though somewhat different phenomenon has been produced by the somnambulist receiving into the mouth something very nasty, and yet pronouncing it to be very agreeable, *under the impression that it was something very nice conveyed by a very dear friend.*" (p. 290.)

That outward impressions will influence the imagination, not only of the somnambulist, but of the ordinary sleeper, and thus give rise to *suggested dreaming*, is sufficiently well-known. We take the following from Lord Brougham's 'Discourse on Natural Theology:'

"The facts upon this subject are numerous, and of undeniable certainty, because of daily occurrence. Every one knows the effect of a bottle of water applied during sleep to the soles of the feet; you instantly dream of walking over hot mould, or ashes, or a stream of lava, or having your feet burnt by coming too near the fire. But the effect of falling asleep in a stream of cold air, as in an open carriage, varies this experiment in a very interesting and striking manner. You will, instantly that the wind begins to blow, dream of being upon some exposed point, and anxious for shelter, but unable to reach it; then you are on the deck of a ship, suffering from the gale, you run behind a sail for shelter, and the wind changes, so that it still blows upon you, you are driven to the cabin, but the ladder is removed, or the door locked. Presently you are on shore in a house with all the windows open, and endeavour to shut them in vain; or, seeing a smith's forge, you are attracted by the fire, and suddenly a hundred bellows play upon it, and extinguish it in an instant, but fill the whole smithy with their blast, till you are as cold as on the road. If you from time to time awake, the moment you fall asleep again, the same course of dreaming succeeds in the greatest variety of changes that can be rung on our thoughts." (p. 112)

And here we think it likely is to be found the key to the *phreno-magnetic* phenomena. In our judgment, no occult influence emanates from the fingers of the mesmerist, and is transmitted, by touching various regions of the head, to the subjacent cerebral organ, exciting the same into unwonted activity; but the head of the somnambulist is touched near the region of presumed organs, and, under some circumstances, the feelings or faculties, associated in the mind of the subject with such locality on the head, are, by suggestion, excited to activity. All who have read of phreno-magnetic doings are acquainted with discoveries of hundreds of organs, just as the training of the patient *in the sleepwaking state* has stimulated the imagination, and led him to the performance of particular acts. On one occasion a gentleman, who was prosecuting experiments upon this subject, and who took our own view regarding the real nature of phreno-magnetism, remarked, in the presence of his somnambulist who was said to be ignorant of phrenology, "now we will try the organ of shaking hands," and then he placed the fingers over the region of destructiveness; immediately the hand was extended for the anticipated purpose. Ten minutes at least having elapsed, and other experiments having intervened, the operator, without a word, touched destructiveness on the opposite side of the head, and the expected result followed, the hand was again extended as before. This subject had been under the manipulations of an itinerant lecturer, who had discovered, by mesmerism, organs for climbing, swimming, excavating, dancing, and a host of others. Some of these discoveries had been made on the head of the person with whom, according to the results obtained by our friend, destructiveness was superseded by a faculty for shaking hands. Dr. Elliotson, however, says that, although *much* comes from suggestion, *all* does not, because he himself only *points* to the organs, making no contact. We refer to Dr. Darwin's case, given in a previous page, which shows that the proximity of warmth will sometimes be appreciated, even should the patient be feelingless to pricking and pinching; and, it may be that, in Dr. Elliotson's instances, the fingers radiate a sufficiency of animal heat to be recognized. Supposing all to be genuine, there is, in some such view of the matter as this, a rational explanation, at any rate, without resorting to the theory of occult influence, or animal magnetism. But again, we are told that results proving mesmero-phrenology have been obtained, where the patients knew nothing either of mesmerism or phrenology, and where, consequently, touch or approach could convey no suggestion. It may be so; but, for the reasons afforded for our scepticism on some other points, we cannot take it upon the evidence. If we had not already extended our remarks so far, and if we did not imagine that our readers would by this time have become somewhat impatient of further detail, we think that we could exhibit a possible source of fallacy in these pretended cases.

In admitting a reality in many of the facts of mesmerism, though rejecting, as unproved, the theory of animal magnetism, we do not offer to explain the rationale of their production. We believe the *modus operandi* to present no uniformity, and to be as yet incapable of a definition that will always apply, just as it is with corresponding affections arising from internal causes. Nevertheless, we conceive the matter to be, as well stated by Dr. Holland in his 'Medical Notes and Reflections,' that "there is no well-authenticated fact making it needful to believe that an influence is received from without, beyond those impressions on the senses, which are capable, according to the temperament and other circumstances, of exciting disordered as well as healthful actions throughout every part of the nervous system, and especially in the sensorial functions." In some instances, the effective antecedent may be a species of fatigue caused by monotony; in others, it may be some unwonted impression on the mind; in many, it may be that sort of mental contagion, sometimes called *imitative sympathy*, well-known to all practitioners who have seen hysteria epidemic in the wards of an hospital, and illustrated to its greatest extent in the history of the dancing mania. In conformity with this latter view, we have been struck with the fact that when and where, in mesmeric investigations, there has been no extended or popular acquaintance with the topic, it has been exceedingly difficult to get a *good subject*; but that, when mesmerism has been seen and heard of by almost every one, subjects in abundance are gained by every experimenter. Thus, in the account of Dr. Sigmond's case, adduced near the commencement of this article, the passages we have marked in italics, exhibit the interest that attached to the topic from previous conversation. We cannot, however, pursue this theme.

We shall not go into any lengthened discussion, respecting the application of mesmerism to the treatment of disease, because, in the first place, we have already attained our permitted limits; and, again, because our object has been rather to examine the validity of reported facts and prevalent theories, than to consider mesmeric doctrines in their practical application. This we hope to have an opportunity of doing hereafter. A very few words must suffice on the present occasion. We must be pardoned if, in the first place, we express our own conviction that, to the great bulk of the reported cases under this head, no importance whatever is to be attached. Every system of quackery that has infested practical medicine from the days of the Asclepiades down to our own times has been supported by abundant proof of this kind; and every individual empiric will boast his successes. We have no superior evidence in favour of the mesmeric cures.

Patients afflicted with certain forms of chronic disease, especially if associated with, or dependent upon defective tone of the nervous system, or depression of spirits, will, it is well known, often recover more rapidly under pleasing stimulation of the imagination and hope, than by treatment which occasions no grateful excitement. Mr. Greenhow, in his published account of Miss Martineau's case, regards the amelioration in the local affection as " the natural sequel of progressive improvement begun in, or antecedent to, the month of April," two months before the employment of mesmerism; this latter, however, he evidently considers to have been conducive, in some degree, towards relieving the concomitant symptoms of nervous distress, as, by a happy coincidence, it was employed just as " the time had arrived when a new and powerful stimulus only was required, to enable the enthusiastic mind of the patient to shake them off:" and thus we take the employment of mesmeric agency to be—purely subjective—in most of the reported examples of its curative power. Some sorts of epilepsy, hysteria, neuralgia, anomalous spasm, chronic dyspepsia, and the like diseases, seem to yield most readily, according to the published accounts, under its use. Sometimes, it may possibly influence certain forms of nervous disease by counter-stimulus, inducing a new and less hurtful condition, and one which may supersede that for which it has been employed: this we advance as a mere suggestion; but, even in this point of view, we suspect its application must ever be limited, from the difficulty of obtaining the artificial state in question, with the great majority of persons. For this latter reason, we fear that operative surgery will never receive extensive benefits from its practice, and that the experience of Madame Plantin, of Wombwell, and some others, will ever continue *extraordinary* cases.

We ourselves have failed to obtain any advantage from mesmerism in several instances where we have employed it, and where we had thought there was room for a fair trial. In our own cases, however, we were careful not to say anything to the patient explanatory of the proceeding, nor did we allude to it as a curative means; and this circumstance suggests a probable cause for the total absence of success.

Were we, however, as a profession, generally disposed to confide in the more rational accounts of mesmeric therapeutics; as we go on, the same shocks to common sense come upon us here, as elsewhere in this inquiry; and then we are thrown back, once more, upon incredulity. Thus we have Mr. Newnham stating, in his recent book, that " In the treatment of many persons together, the magnetisation of trees, or other inanimate substances, may be useful: and the facts are not inconsistent with the general laws of magnetism." We have, moreover, scientific and erudite men gravely proclaiming somnambulists to be the surest prescribers for diseases, and maintaining that practitioners should hold them in readiness, as guides and directors in the management of obscure cases: And the British metropolis contains at least one physician who indulges in these lamentable extravagances! Of Dr. Elliotson we would not speak but with unfeigned regard. He has our sincerest esteem for the services he has rendered to practical medicine; and, beyond his high qualifications as a physician and scholar, there is a boldness and directness of purpose in his proceedings which we love to see. He, himself, will not respect us the less, because we decline to follow him blindly, or any other individual, however estimable.

We have accomplished our task. We have striven to realize the intention expressed at the outset of this article, and have, to the best of our ability, given to mesmerism a fair, candid, and certainly unprejudiced consideration. In the prosecution of this design, we have been swayed by no fears that we might bring upon ourselves the wrathful stripes of the more ardent and enthusiastic of its votaries; nor, on the other hand, have we dreaded the ridicule of some of our brethren, in declaring a full belief in the reality of some of the facts, often set down as sheer delusion or imposture; nor been anxious to gain for ourselves that species of security often anticipated by those who cautiously take the *via media.* We have examined the subject purely as a matter of evidence, qualified by our own observations and reflections; and, in taking leave of the subject, we shall briefly recapitulate the conclusions at which we have arrived.

We conceive, then, that making abstraction of all the roguery and deceit so prevalent in mesmeric proceedings, there is a reality in some of the facts: that these consist of certain forms of nervous disorder often arising spontaneously under the vague designations of hysteria and somnambulism; that in these, when induced by mesmerism, there is no speciality except in the mode of their origin; and that they are brought about, not by any distinct agent as implied by the term *animal magnetism*, but by peculiar impressions made on the organs of sense and the inward consciousness. We conceive, moreover, that whilst we may fairly speak of mesmerism as a new *art*, evidence is wanting to show that it is a new *science*, involving any principle hitherto unknown. For the same reason, defective proof, we reject the reported phenomena, implying the receipt of intelligence through any other media than the customary organs of sense; although, in our judgment, it were better calmly to investigate these alleged marvels than rudely to deride them. This is a principle of action by which we have ever been guided. We hold ourselves in readiness to witness and candidly to examine any novel fact that may come in our way, as we believe our minds to be open to conviction on satisfactory evidence being adduced; and, whilst we have at all times pursued this method ourselves, it is the one we would, in conclusion, earnestly recommend to our readers.*

* Since the above was in type, we have been much gratified by perusal of an article on mesmerism, in the last (February) number of Blackwood's Magazine. The author of the paper has evidently given to the subject much consideration, as well as some personal investigation. He examines the entire question, free alike from the shallow contempt of one party, and the preposterous credulity of the other. A truth in mesmerism is recognized, however commingled with error and absurdity; and the attempt at its discovery is judiciously made. The production very naturally commends itself to our approval, as some of the writer's views and conclusions coincide very much with those to which we have ourselves come; but, independently of this, there is a soundness in the whole tone and spirit which communicates to it an uncommon degree of excellence. Certain *physical* objections to the possibility of clairvoyance are put in a manner at once striking and novel. We strongly recommend the article to the attention of our readers.

