# Dr. Elliotson and Mesmeric Diagnosis

**Published:** 1845-07

**Authors:** 


### DR. ELLIOTSON AND MESMERIC DIAGNOSIS.

In the article on Mesmerism, in our last Number, the reader will find at p. 484, the following statement: "We have, moreover, scientific and erudite men gravely proclaiming somnambulists to be the surest prescribers for diseases, and maintaining that practitioners should hold them in readiness, as guides and directors, in the management of obscure cases: and the British metropolis contains at least one physician who indulges in these lamentable extravagances:" and it was implied that Dr. Elliotson was the physician alluded to. We have since learned, on the best authority, that of Dr. Elliotson himself, that we were entirely mistaken in making this statement; and we now express our sincere regret for the error we committed in so doing. Our mistake originated in the first place, in the misinterpretation of a passage in his address to the Phrenological Association in the summer of 1843, and published in the October number of the Zoist of the same year; and our mistaken impression seemed to be confirmed by the subsequent detail of certain cases communicated to Dr. Elliotson and published by his authority in the Zoist, in which the power of a somnambulist to discriminate and prescribe for disease is, in several instances, attested. It gives us sincere pleasure to know that Dr. Elliotson does not himself "indulge in these lamentable extravagances;" and that, in publishing the cases referred to he merely intended to record the statement of others with a view to promote inquiry.

